# The development and preliminary validation of a multidimensional assessment scale for loneliness in nursing homes

**DOI:** 10.1016/j.ijnsa.2026.100685

**Published:** 2026-09-24

**Authors:** Dick van de Kolk, Robbert Huijsman, Dirk Frans de Korne, Gerda Andringa

**Affiliations:** a Erasmus School of Health Policy & Management, Erasmus University Rotterdam (ESHPM, EUR), Postbus 1738, 3000, DR, Rotterdam, the Netherlands; b Stichting voor Regionale Zorgverlening Zeeland (SVRZ), Postbus 100, 4330, AC, Middelburg, the Netherlands; c University College Roosevelt (UCR) Middelburg, Lange Noordstraat 1, 4331, CB, Middelburg, the Netherlands; d Academische werkplaats voor ouderenzorg Zeeland (AWOZ), the Netherlands

**Keywords:** Multidimensional loneliness, Nursing homes, Cognitive impairment, Scale development, Cognitive interviewing

## Abstract

**Background:**

Loneliness among nursing home residents is a multidimensional and subjective experience associated with adverse health outcomes. Existing instruments often fail to capture this complexity and are poorly adapted to cognitively impaired populations in institutional care settings.

**Objectives:**

To create a multidimensional, cognitively accessible assessment scale for loneliness in nursing home residents, grounded in residents’ experiences and suitable for use in routine care.

**Design:**

Sequential multi method study following the initial stages of scale development as outlined by Boateng et al. (2018).

**Settings:**

Multiple nursing home settings in the Netherlands represent long-term care environments for frail older adults.

**Participants:**

Nursing home residents were purposively selected to reflect variation in cognitive functioning, including individuals with no cognitive impairment, mild cognitive impairment, and moderate to severe cognitive impairment. Participants were included if they could engage in an interview to some extent; residents with acute distress or an inability to participate in any form of communication were excluded**.**

**Methods:**

Item development followed three steps: (1) generation of items and recommendations based on literature and residents’ experiences; (2) expert consultation to establish content validity and refine wording; and (3) pre-testing through semi-structured cognitive interviews (Willis, 2004) to assess comprehension and feasibility.

**Results:**

The final scale comprises 14 items selected from an initial pool of 22. The instrument captures social, emotional, existential, and institution-specific dimensions of loneliness. Although comprehension was lower among residents with severe cognitive impairment, overall understanding across participants was sufficient to retain all items. Cognitive testing demonstrated that residents, including those with moderate to severe dementia, were able to meaningfully reflect on the items when appropriately supported. The assessment procedure was found to be practical, inclusive, and minimally burdensome, and facilitated discussion of residents lived experiences.

**Contribution of the paper**

What is already known about the topic?

• Loneliness in nursing home residents is a subjective, multifaceted experience that is difficult to assess, particularly in those with cognitive impairment.
• Existing instruments often lack conceptual scope and are not sufficiently adapted to the cognitive and contextual realities of nursing home populations.
• There is a need for feasible, low-burden assessment methods that capture multiple dimensions of loneliness in institutional long-term care settings for elder adults.

What this paper adds

• A 14-item multidimensional loneliness scale tailored for nursing home residents, capturing social, emotional, and existential dimensions while incorporating institutional aspects.
• Evidence that residents with moderate to severe dementia can meaningfully engage with a multi-item loneliness assessment when supported by appropriate methods.
• Demonstration that the instrument is practical, inclusive, and minimally burdensome, and can support reflective dialogue on residents lived experiences when administered by trained staff, thereby contributing to both research and person-centred care.

## 1 Introduction

Older adults are particularly vulnerable to loneliness due to age-related changes, including declining physical health, functional limitations, contraction of social networks, and the loss of significant social roles, relationships, and meaningful activities (Lim et al., 2020; Sullivan et al., 2016; Van Campen, 2018). This risk appears to increase in nursing homes, where the transition to residential care may exacerbate feelings of isolation and disconnection (Gardiner et al., 2020; Victor, 2012; Salari et al., 2025). Loneliness is defined as “the subjective experience of an unpleasant or intolerable lack of (quality of) certain social relationships” (van Tilburg and de Jong-Gierveld, 2007). It is a complex and multifaceted phenomenon that encompasses a wide range of causes and manifestations (De Jong-Gierveld and Van Tilburg, 2016; Yanguas, 2018). Loneliness is associated with serious and harmful effects, on health and well-being and has been linked to a range of adverse outcomes among nursing home residents, including depression, accelerated cognitive decline, and premature mortality (e.g. Livingston et al., 2024; Lapane et al., 2022).

Nursing home residents may experience loneliness differently from older adults living in the community, including unmet needs for social companionship, the absence of close emotional bonds, and profound feelings of emptiness, meaninglessness, or existential isolation (Barbara et al., 2019; Paque et al., 2018; Sundström et al., 2019). Given this diversity of manifestations, a multidimensional approach incorporating social, emotional, and existential dimensions appears most appropriate for capturing loneliness among nursing homes residents (Greenwood et al., 2018; Zhang et al., 2023; Jansson et al., 2022). Social loneliness arises from a perceived lack of meaningful social relationships, whereas emotional loneliness stems from the perceived absence of close and intimate attachments (De Jong-Gierveld and Van Tilburg, 2010). Existential loneliness is generally understood as a profound sense of fundamental separateness from others and the world and has been associated with experiences of frailty, serious illness, dependency, awareness of mortality, and proximity to the end of life (Ettema et al., 2010; Bolmsjö et al., 2019). Together, these dimensions provide a comprehensive framework for understanding loneliness in later life and are widely recognised as distinct yet related constructs within contemporary loneliness research (Zhang et al., 2023; Schoenmakers and Tromp, 2022; Jansson et al., 2022). However, loneliness in institutional settings may also be shaped by contextual factors that extend beyond social, emotional and existential dimensions. Nursing homes provide long-term residential living, 24-hour supervision, assistance with daily activities, and ongoing healthcare support, often for individuals with substantial physical or cognitive impairments (Sanford et al., 2015). Consequently, residents are confronted not only with age-related losses but also with the challenges of adapting to a highly care-dependent living environment, including reduced autonomy, changes in personal identity and roles, separation from previous social environments, and increasing dependency on others (Paque et al., 2018). As a result, social, emotional, and existential loneliness should be viewed as important but potentially non-exhaustive dimensions of loneliness in nursing home populations. This underscores the need for assessment approaches that are sensitive to the distinctive ways in which loneliness may be experienced and expressed within residential nursing home care, particularly among individuals with dementia (Kotwal et al., 2024).

A range of measures have been developed to assess loneliness among older adults, from single-item indicators to multidimensional scales (Bugallo-Carrera et al., 2023; Maes et al., 2022). Given the inherently subjective nature of loneliness, assessment instruments are typically designed to capture individuals' own experiences and perceptions (Maes et al., 2022; Van Tilburg and De Jong-Gierveld, 2007). As a result, loneliness is most assessed using self-report measures, although proxy assessments by relatives or caregivers are sometimes used when self-report is considered difficult or infeasible (Griffiths et al., 2020). However, proxy reports may not fully reflect the subjective experience of loneliness and can diverge from individuals' self-reported perceptions (Victor et al., 2005). While self-report measures may be appropriate for nursing home residents with sufficient cognitive and communicative abilities, their application becomes more challenging among residents with dementia, for whom impairments in memory, language, and comprehension may affect the accurate reporting of subjective experiences (Smebye et al., 2012). Assessing loneliness in this population therefore requires balancing the limitations of both approaches: self-report may be constrained by cognitive and communicative impairments, whereas proxy assessments may not fully capture residents' internal experiences of loneliness. Additionally, the presence of others during assessment may unintentionally influence responses through subtle verbal or non-verbal cues further complicating the measurement of loneliness in residents with dementia (Smebye et al., 2012; Bender et al., 2022). Considering these measurement complexities, loneliness in nursing home settings is sometimes assessed using a single direct question such as: “Do you sometimes feel lonely” (Jansson et al., 2017; Drageset and Eide, 2020). While pragmatic and easy to administer, such approaches may not fully capture the multidimensional and context-dependent nature of loneliness experiences among nursing home residents. More widely applied are multi-item measures that assess loneliness through indirect questions, thereby explicitly avoiding the term ‘lonely’ (Bugallo-Carrera et al., 2023; Newmyer et al., 2021). Examples include the Revised University of California, Los Angeles (R-UCLA) Loneliness Scale (Russell et al., 1980) and the Jong-Gierveld Loneliness Scale (de Jong-Gierveld and Kamphuis, 1985) which is based on a multidimensional conceptualization of emotional and social loneliness. Over time, several versions of both scales have been developed, including abbreviated forms for use in older and more vulnerable populations (Bugallo-Carrera et al., 2023). Although these instruments widely have been used among older adults, their application in nursing home populations has been associated with several challenges, including high attrition rates and difficulties completing assessments or providing informed consent due to cognitive impairment Jansson et al., 2017; Nyqvist et al., 2013; Mann et al., 2020).

Several conceptual and methodological complexities continue to hinder the accurate assessment of loneliness among nursing home residents (Maes et al., 2022; Wong et al., 2022). These challenges are outlined below and correspond to the process diagram shown in Fig. 1. First (C1); most existing tools conceptualise loneliness as a stable condition, despite evidence that loneliness can be also a momentary condition (Zhaoyang et al., 2022). In nursing homes loneliness may fluctuate across situations, social contexts, and daily routines (Zhang et al., 2023). This context-dependent nature of loneliness suggests that assessment outcomes may be influenced by measurement conditions, such as the timing and setting of the assessment, the mode of administration, and the extent to which information from caregivers is incorporated. Accordingly, further research is needed to identify optimal conditions for the reliable assessment in nursing home populations. Second (C2); self‑report scales may be less suitable for a substantial proportion of residents, given age‑related declines in vision, cognitive functioning, attention, and concentration (Mann et al., 2020; Verbeek-Oudijk and Koper, 2021 p.21.). Moreover, reliance on self-report measures may introduce response bias, as residents might fear being perceived negatively or being stigmatized, they may underreport their loneliness (Barbara and Petersen, 2025). Third (C3); most tools were developed and validated among older adults living in the community, raising concerns about their cultural and contextual relevance when applied in institutional settings (Victor, 2012). To address these limitations, researchers recommend modifications such as simplified language, visual aids, and observational methods to complement assessment scales, especially for individuals in advanced stages of dementia (McConnell et al., 2019; Murphy et al., 2015; Victor, 2012). Fourth (C4); the cognitive and linguistic demands of many multi-item instruments may reduce comprehensibility and increase response burden among residents with cognitive impairment, underscoring the need for simplified assessment formats (Smebye et al., 2012; Chapman et al., 2024; Ayton et al., 2021).

**Fig. 1:**
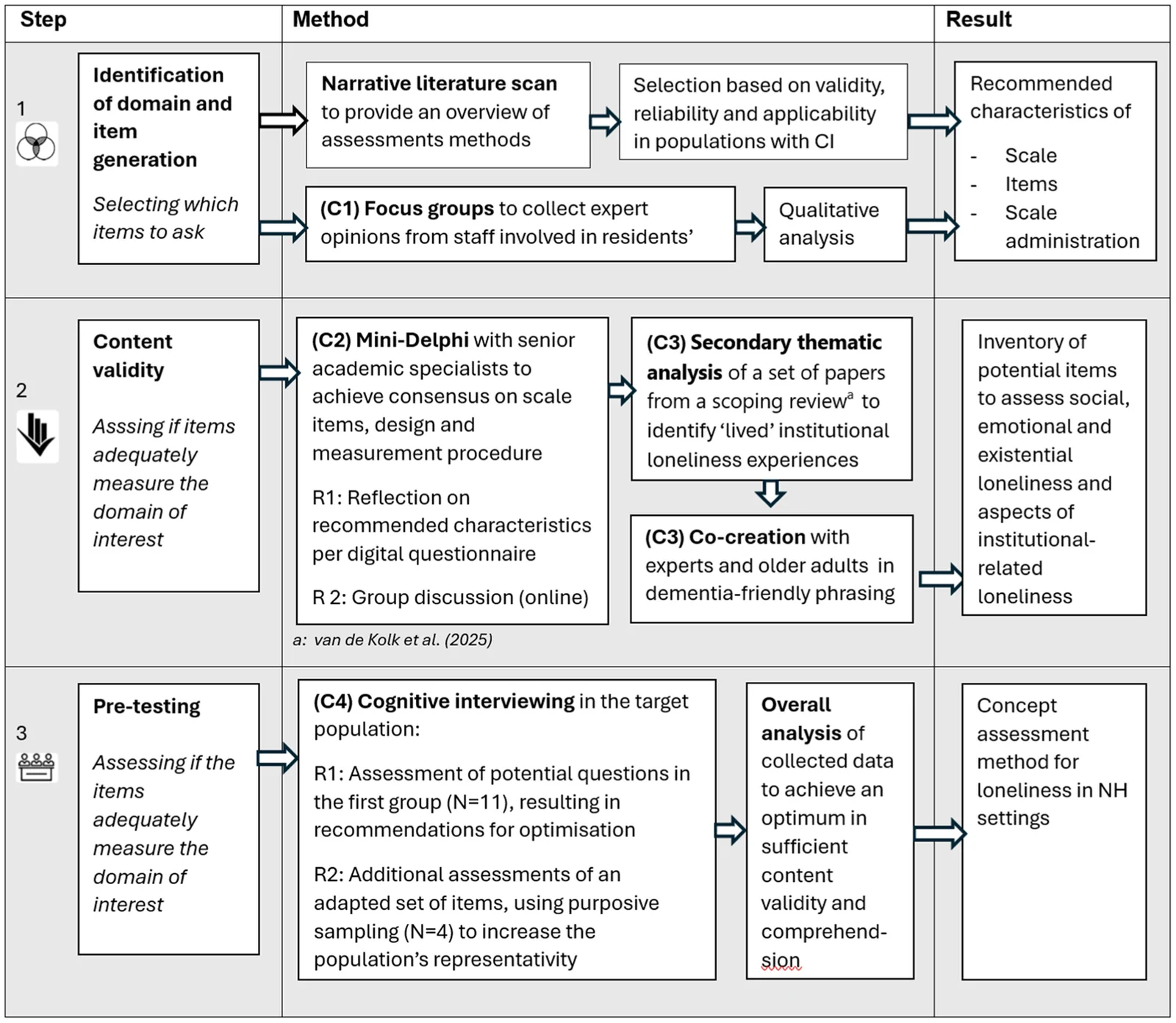
Procedure for scale development (Boateng, 2018). The numbered methods correspond to the conceptual and methodological challenges: (C1) measurement conditions, (C2) Response bias (C3) Institutional context (C4) Cognitive capacities.

To achieve a comprehensive understanding of loneliness-related needs and experiences in nursing home settings, it is essential to include individuals with advanced cognitive impairments in both cross-sectional and longitudinal research (Gardiner et al., 2020). This is particularly important given their expected steadily increasing representation within nursing home populations (Mukamel et al., 2023; Alzheimer's Association, 2016). Addressing this gap necessitates the development and implementation of customised assessment instruments that adequately capture the multidimensional nature of loneliness as experienced by this group (van de Kolk et al., 2025; Bugallo-Carrera et al., 2023). By adopting approaches that reduce participation barriers and capitalize on the retained strengths of individuals advanced cognitive impairments, the multidimensional experience of loneliness can be captured more accurately and comprehensively (Shannon et al., 2021; Cridland et al., 2016; O’Shea et al., 2025). Building on these insights, the present study aims to develop a scale for multidimensional measurement of loneliness among older adults (≥60 years) residing in nursing homes and receiving long-term care. The proposed instrument will explicitly incorporate residents lived experiences and will be tailored to the abilities of individuals with advanced cognitive impairments. By doing so, it seeks to enhance the validity and sensitivity of loneliness assessment in institutional care contexts, ultimately facilitating the development of more targeted and effective interventions within nursing home settings.

## 2 Method

### 2.1 Overall study design

This study employed a sequential multi-methods design and was conducted across 11 nursing home settings within three long-term care organisations in rural region in the Netherlands. Together, these organisations provide care for a diverse population of older adults with somatic, psychogeriatric, and complex care needs. Instrument development was guided by the framework proposed by Boateng et al. (2018), which was adapted to the aim of the present study. Although the complete framework comprises nine steps across three phases of item generation, scale development, and psychometric evaluation, the present study is limited to the first three steps within the initial two phases. Accordingly, this study focused on construct exploration, item development, and scale refinement using cognitive interviewing techniques (Willis, 2004), thereby addressing the four key limitations of the existing loneliness questionnaires identified in the introduction (Fig. 1, C1-C4).

### 2.2 Step 1: Item generation

The process started by defining the domain, key constructs, and the scope of the scales. Subsequently, a narrative literature review was conducted to identify existing loneliness measures and examine how loneliness could be assessed among nursing home residents. The primary objective was to identify existing validated loneliness instruments that adequately reflected the social, emotional, and existential dimensions of loneliness and could inform the development of the adapted instrument. Searches were performed in Google Scholar, PubMed Central, APA PsycInfo, and ScienceDirect using combinations of the terms: loneliness, loneliness scale, older adults, nursing home, long-term care, LTC, and dementia. The search process was iterative and supplemented by snowball sampling through screening the reference lists and citations of relevant publications to identify additional studies and measurement instruments. In addition to peer-reviewed literature, grey literature sources were considered, including doctoral dissertations and research reports. Studies published in Dutch or English were screened based on title and abstract for relevance to loneliness assessment in older populations and long-term care settings. Data extraction focused on the characteristics of identified measures, including their conceptual domains, target populations, scale characteristics (e.g., length and wording), and evidence of application in nursing home settings. Measures assessing social and/or emotional loneliness were eligible if they had been frequently applied and psychometrically evaluated among older adults. Given the limited representation of existential loneliness measures, eligibility was based on prior application among older adults. As this was a narrative rather than a systematic review, no formal quality appraisal was undertaken.

Following Morgan’s (2019) focus group methodology, focus groups with nursing home staff were conducted to explore the feasibility of assessing residents’ loneliness. Two focus groups were deemed sufficient for the exploratory phase of instrument development, as the aim was to identify key concepts and capture a broad range of perspectives rather than achieve full thematic saturation (Hennink et al., 2019). A purposive sampling approach was used to recruit 17 participants, who had on average 6.5 years of experience, for two focus groups (Table 1). Participants were selected from three nursing home organisations and represented a range of roles, including nursing and care staff involved in daily resident care, well-being staff responsible for social and recreational activities, and volunteers providing informal social support and companionship.

**Table 1:** Demographic information of the participants of focus groups.

Nr.	Organi-sation	Function	Care unit	Experience^B^ (y)
D1.1	A	Nurse Practiconer (NP)	PG/Som^A^	2
D1.2	B	Nursing assistant (Dutch Verzorgende IG)	PG	15
D1.3	B	Nursing assistant (specialized in psychogeriatric care)	PG	10 (2)
D1.4	A	Nurse	PG/Som.	23
D1.5	A	Nursing assistant (Verzorgende IG)	Som.	5
D1.6	B	Nursing assistant in training	PG/Som.	3
D1.7	C	Well-being coach	PG/Som.	10
D1.8	B	Physician	PG/Som.	2
D2.1	C	Well-being coach	PG	0.5
D2.2	B	Spiritual caregiver	PG/Som.	9
D2.3	A	Residential Support Worker	PG/Som.	0.5
D2.4	B	Residential Support Worker	Som.	5.5
D2.5	B	Wellbeing coach	PG/Som.	4
D2.6	A	Residential Support Worker	Som.	1.5
D2.7	B	Volunteer well-being	PG	1
D2.8	B	Well-being worker	PG	14
D2.9	C	Behavioural support coach	PG/Som.	2

Note:.

A: PG = Wards for patients with Psychogeriatrics needs; Som. = Wards for Patients with somatic needs.

B: Years of experience with the target population.

In addition to gathering recommendations on item content, the focus groups aimed to identify strategies for administering the scales to residents with dementia. To ensure consistency and procedural standardization across focus groups, a predefined protocol was used (supplementary materials 1, p4). This included general, inductive questions about the measurability of loneliness in nursing home contexts, complemented by a deductive component in which participants discussed the measurability of specific items in their routine work. Finally, audio recordings of focus groups were transcribed verbatim with minor readability adjustments and anonymisation by two assistant researchers. Following transcription, thematic analysis was conducted by a research-assistant using a combined deductive–inductive approach (Fereday and Muir-Cochrane, 2006). Deductive coding was guided by predefined categories discussed in the focus groups, while inductive coding integrated participants’ own phrasing. Codes were iteratively refined and organised hierarchically into themes and categories (Supplementary Materials 1, p8). Data were managed and analysed using NVivo Release 1.7 (Lumivero, Denver, CO, USA). Finally, theses analysis identified dominant themes aligned with the three-dimensional conceptualisation of loneliness. These findings guided the initial prioritisation of candidate items derived from the literature and provided a first exploration of key considerations regarding the administration of the assessment.

### 2.3 Step 2: Content validity

First, expert consensus was sought through a two-round mini-Delphi process to ensure that the emerging assessment adequately reflected the multidimensional nature of loneliness while remaining feasible for use among people with dementia. The mini-Delphi approach facilitated a structured consensus process within a small expert panel and limited timeframe (Keeney et al., 2011; Schoeber et al., 2022). The panel comprised five Dutch scientific experts, two specialised in loneliness, and three specialised in care ethics. All participants of the mini-Delphi holding doctoral degrees, including one full professor, valuated whether the themes identified in the focus groups adequately represented social, emotional, and existential loneliness in nursing home residents. In the first Delphi round, experts were asked to map candidate items to corresponding items from existing loneliness measures and identify areas requiring additional content. Feedback was also sought on practical aspects of instrument administration, including proxy involvement, response formats, and the assessment timeframe. While predefined consensus criteria were applied, each topic was accompanied by an open-text field. As these comments frequently provided richer insights into conceptual relevance, item appropriateness, and feasibility than the ratings themselves, subsequent refinement was informed by both quantitative and qualitative feedback (Diamond et al., 2014). In the second mini-Delphi round, four experts from the initial panel engaged in a structured consensus process to refine and prioritise candidate items and further develop the conceptual framework and measurement approach, consistent with recommendations for Delphi-based instrument development (Keeney et al., 2011).

Building on the findings of the mini-Delphi process, a secondary thematic analysis was conducted using qualitative studies from the full-text dataset of a previously completed scoping review by van de Kolk et al. (2025). Seven studies that investigated the lived experiences of loneliness among nursing home residents were included (Jansson et al., 2022; Huang et al., 2022; Amzat and Jayawardena, 2016; Barbosa Neves, Barbara et al., 2019; Paque et al., 2018; Chung et al., 2020; Sundström et al., 2019; Österlind et al., 2017). Using in vivo coding and directed content analysis (Hsieh and Shannon, 2005), reported experiences were first mapped to items from existing loneliness measures. Experiences not represented in these measures were subsequently inductively clustered into institution-specific themes, which informed the development of additional candidate items. Subsequently, through a co-creation process comprising three stages, the items were transformed into appropriate questions. First, a multidisciplinary expert group, consisting of relevant academic experts (two speech-language therapists, an expert in cognitive ergonomics and artificial Intelligence, and a policy advisor in social gerontology and care ethics), reformulated items from the original self-assessment scale into simplified, and accessible (Dutch) language. A tiered approach was developed, enabling items to be supported by concrete examples in words and in images. However, the use of images was ultimately discarded due to their potential for multiple interpretations. Second, in accordance with contemporary recommendations for minimizing acquiescence bias through the use of balanced item wording (e.g. Martins et al., 2023), we incorporated both negatively and positively phrased items, consistent with the structure of the original 11-item De Jong Gierveld Loneliness Scale (de Jong-Gierveld and Kamphuis, 1985) and the Existential loneliness questionnaire (Mayers et al., 2002). The response format was further elaborated by drawing upon the psychometric design of a psychogeriatric mood assessment instrument (Diesfeldt, 1997) and aligned with the methodological approach described by Wong et al. (2022). Each item consisted of a key concept accompanied by a brief interrogative statement and was rated on a three-point Likert-type response scale ("yes", "more or less", and "no"). Simplified response formats have been shown to improve comprehensibility and feasibility in dementia research (Chapman et al., 2024; Ayton et al., 2021), while the intermediate response option allows respondents to express nuanced experiences of loneliness without requiring more complex distinctions between multiple response categories. Finally, the revised items were tested by a panel of five older adults recruited through a network for older adult participation and a day care centre to assess their clarity and comprehensibility. The purpose was to assess the appropriateness, clarity, and relevance of the language for older adults and to ensure that the items resonated with their experiences. Feedback was reviewed by the research team and integrated into the final version of the scale.

### 2.4 Step 3: Pre-testing

We tested a set of scale items collected in stage two for meaningfulness and comprehension among subjects from a representative population.

#### 2.4.1 Study design

Data were collected from a purposively selected sample of participants using a structured cognitive interviewing approach to evaluate how individuals from the target population understood, interpreted, and formulated responses to each questionnaire item. This assessment focused on four cognitive processes involved in answering survey questions: comprehension of the item, retrieval of relevant information from memory, evaluation and judgment of the retrieved information, and formulation of a response (Willis, 2004). Cognitive interviews are typically conducted using the think-aloud technique, in which respondents verbalize their reasoning while answering questions. However, this approach may impose substantial cognitive and communicative demands, particularly on individuals with limited verbal or cognitive abilities, such as those included in this study. Therefore, verbal probing, a recommended alternative, was employed. This method involved asking simple, targeted questions to elicit information about participants’ responses. In accordance with Willis (2004), multiple rounds of cognitive interviews were undertaken, with iterative modifications implemented between rounds to enhance question comprehension and interpretability

#### 2.4.2 Participants and recruitment

Recruitment was facilitated through designated contact persons within each participating organisation, who distributed study invitations and supported the informed consent process. In accordance with ethical standards and legal requirements, informed consent was obtained through a carefully structured process conducted by a familiar staff member, using a personalised and flexible approach (Roelofs et al., 2021; Thorogood et al., 2018). Participants with legal capacity provided written informed consent; for individuals lacking capacity, consent was obtained in written form from an authorised representative (e.g., a legal mentor, appointed or health care proxy). Across the rounds, 10 to 15 participants were recruited. This is consistent with methodological guidance suggesting that 5 to 15 interviews per round are typically adequate to uncover most problems related to question understanding, retrieval, judgment, and response formulation (Blair and Conrad, 2011; Willis, 2004). To achieve a sufficient sample size representative of the intended population, convenience and purposive sampling methods were combined, based on sampling data from the Dutch National Statical Office (Centraal Bureau voor de Statistiek, 2023). In addition, eligibility criteria were applied to ensure appropriate participation and the quality of the collected data. Exclusion criteria for the pre-test sample included severe communication disorders, psychiatric conditions, reduced awareness, advanced cognitive impairment and an assessed high risk of behavioural and psychological symptoms of dementia (BPSD) that could interfere with participation in the study procedures. Cognitive status was pre-assessed by staff, using the Dutch translation of the Global Deterioration Scale (GDS, Reisberg et al., 1982) which ranges from normal cognition (stage 1) to very severe decline (stage 7). Participants with a Global Deterioration (GDS) stage of 7, indicative of advanced dementia were excluded because were excluded because meaningful participation in the interviews was no longer feasible (De Jong et al., 2005). All participants residing in psychogeriatric departments had a Global Deterioration Scale (GDS) stage of 5 or 6 and a documented psychogeriatric diagnosis, including dementia in general (n = 2), vascular dementia (n = 1), Alzheimer's disease (n = 3), and Lewy body dementia (n = 3). A total of 15 participants were found eligible, deemed capable of completing the 60-minute cognitive interviews and answering straightforward questions (Table 2). Pre-existing complaints included: speech difficulties due to incorrect breathing (1), aphasia (3), hearing impairment (1), and memory problems (1).

**Table 2:** Participant characteristics.

Characteristic	Round 1 (N = 11)	Round 2 (N = 4)	Overall (N = 15)
Age (range 62–92)	83	84	83
Gender (male)	5	3	8
Residence on psychogeriatric ward	7	4	11
GDS score^a^ 1–4 (no – mild cognitive decline)	3	0	3
GDS score^a^ 5 (moderate – severe cognitive decline)	5	1	7
GDS score^a^ 6 (severe cognitive decline)	2	3	5

Note:.

a: Global Deterioration Scale (Reisberg et al., 1985).

#### 2.4.3 Data collection

Cognitive interviews were conducted using a structured interview protocol based on Willis (2004) and were limited to a maximum of 60 min. The protocol included standardized instructions for respondent recruitment and contact, the informed consent procedure, and interview completion (Supplementary Materials 1, p24). Items were presented in random order to compensate for expected incomplete assessments and to ensure that all items received approximately equal evaluation across participants. Following each item, participants were requested to select the response option that best reflected their preference, together with (1) a general question “Did you find it a difficult or easy question?”, (2) a comprehension probe "What does the main 〈phrase of the main concept〉 mean in this question?" and (3) a recall probe “When did you experience (bolded phrasing of concept in question)?” Verbal responses and field notes were documented via audio recordings and in a structured form.

For the first two pilot interviews, the primary researcher and a research assistant were both present on site and independently documented observations and conducted clinical assessments. The remaining interviews were conducted by the primary researcher in two rounds, after which the research assistant independently reviewed the audio recordings. Both assessors independently evaluated responses according to the criteria outlined in Table 3. In this set-up and in accordance with the Landis and Koch (1977) classification, the linearly weighted Cohen’s kappa (κ = 0.76) indicated substantial agreement (supplementary materials 2).

**Table 3:** Comprehension assessment score.

Rate	Comprehension	Observation
1	Absent	Lack of response or a response that clearly indicates a lack of comprehension or ability to express
2	Insufficient	Difficult to assess, or a response that indicates insufficient comprehension or expression of the response
3	Partial	Largely assessable and a response indicating at least a partial adequate comprehension and expression of the response
4	Sufficient	Sufficiently assessable and sufficient indication of comprehension and expression of the response.
5	Convincing	Complete, accessible, and convincing comprehension and expression of the response.

#### 2.4.4 Data analysis and questionnaire revision

Data analysis followed an iterative, multi-step process. It began with the examination of four pilot interviews, during which the primary researcher, an assistant, and a senior researcher, drawing on the first interviews and field notes, collaboratively discussed evaluation criteria for each question to establish common assessment standards. Second, after each successive interview round, the researchers independently compared and discussed item scores, ranking from 1 to 5 per respondent to establish a final item comprehension score. The primary researcher resolved conflicting ratings by re-listening to the recordings and reviewing the relevant field notes to assign a final score. Third, after the second round, an indicative average comprehension score was calculated across all participants, with a threshold of four indicating adequate understanding. The evaluation of items, rephrased between test rounds, was primarily based on the average comprehension score of participants who received the modified version. Finally, the research team reviewed and finalised the questionnaire, ensuring that respondents’ comprehension was optimised while preserving maximal conceptual alignment with the original scales across all dimensions.

## 3 Results

This section presents the findings for each applied step of Boateng and colleagues’ framework (2018), with an integrated overview provided in Table 4. The items retained after the evaluation are listed in the final column, “Approved.”

**Table 4:** Results item-development per step.

No^A^		Step 1 Identification of domain and item generation		Step 2 Content validity	Step 3 Pre-testing					Ap-pro-ved
		Scientific input^B^	Staff opinion	Adapted phrasing (translated from original Dutch version ^F)^	R1^G^	R2^H^	N	Ind =>4^I^	Score^J^	
1	**Social dimension**	DJGLS 1	-	*If you have daily problems, are there always people you can talk to?*	4	-	10	80%	-	-
2		DJGLS 4^C^	-	*If you experience bad things, are there enough people who support you? ^D^*	4,1	3,7	14	64%	3.9	✓
3		DJGLS 7^C^	-	*Are there many people you can completely trust?*	4,4	4	14	92%	4.3	✓
4		DJGLS 8^C^	Lack of social con-nectedness	*Are there enough people with whom you have a close relationship?*	4,4	4,3	13	100%	4.4	✓
5		DJGLS 11	-	*You can turn to your friends if you think you need them?*	4,4	-	9	100%	-	-
6	**Emotional dimension**	DJGLS 2	Longing for close relationships	*Are you missing a close friend?*	3,7	-	7	71%	-	-
7		DJGLS 3^C^	-	*Do you experience a sense of emptiness around you?*	4,2	3,8	15	64%	4,1	✓
8		DJGLS 5	-	*Do you miss the pleasure of the company of others?*	4,1	-	10	80%	4,1	-
9		DJGLS 6	-	*Do you think your circle of acquaintances is too limited?*	4,5	-	10	90%	-	✓
10		DJGLS 9^C^	-	*Do you miss people around you?*	4,4	3,3	11	64%	4,1	-
11		DJGLS 10^C^	-	*Do you often feel left behind?*	4,4	4	12	83%	4,3	✓
12	**Existential dimension**	ELQ 1	-	*Are you happy with how you have lived?*	3,3	-	10	44%	3,5	
				*Do you look back on a happy life? ^E^*	*-*	4	4	100%		✓
13		ELQ 3	-	*Does your life have a purpose?*	3,8	-	10	80%	3,9	
				*Does life give you fulfilment?*	*-*	4	4	100%		✓
14		ELQ 18	Helplessness	*Do you feel helpless?*	4,2	4,3	14	85%	4,2	✓
15		ELQ 25	-	*Do you feel powerless in the face of the world?*	3,7	-	7	86%	3,6	-
				*Do you feel that you have little to decide for yourself here? ^E^*	-	3.5	4	50%		
16		ELQ 26	-	*Do you feel dead inside?*	3,5	-	8	63%	3,7	
				*Do you often think “life doesn't have to be like this for me anymore”? ^E^*	-	4	4	75%		✓
17		ELQ 27	-	*Does this world offer many possibilities?*	3,6		8	63%	3,8	
				*Are you still able to do the things you enjoy doing? ^E^*		4	3	75%		✓
18		ELQ 29	Meaning-lessness	*Does life have little meaning for you?*	4,1	4,3	13	92%	4.2	✓
19	**Institutional aspects**	IAL 1		*Have you lost your own identity?*	3,9		10	70%	3,8	-
				*Can you be yourself here? ^D^*		3,5	4	50%		
20		IAL 2		*Do you feel attached to this place?*	4,7		10	100%	4.5	
				*Do you feel at home here? ^E^*		4,3	4	100%		✓
21		IAL 3		*Do you often feel nostalgic for the past?*	4,5	3,3	13	75%	4,1	-
22		IAL 4		*Do you feel excluded?*	4,4	3,8	13	75%	4.2	✓

Note:.

A: Number in 22 item test-set.

B: DJGLS: De Jong‑Gierveld Loneliness Scale (de Jong-Gierveld and Kamphuis, 1985); ELQ: Existential Loneliness Questionnaire (Mayers et al., 2002); IAL: Institutional Aspects of Loneliness.

C: item corresponding with 6-item De Jong‑Gierveld Loneliness Scale (De Jong-Gierveld and van Tilburg, 2006).

D: rephrased based on round 1 evaluation based on phrases in cognitive interviews round.

E: rephrased based on round 1 evaluation to improve semantic understanding in moderate dementia.

F: Original Dutch version of 22 test items, see: supplementary materials 1, p22.

G: Mean Global Deterioration Scale score of first round participants (n=11): 4.2,

H: Mean Global Deterioration Scale score of second-round participants (n=4): 5.8).

I: Percentage of individual scores that meet the standard of sufficient comprehension (score =>4).

J: Average item comprehension score over all participants in round 1 and round 2.

### 3.1 Step 1: Item generation

First, the narrative review indicated that loneliness assessment in nursing homes generally relies on three approaches: validated loneliness scales, direct self-report questions, and qualitative explorations of residents' loneliness experiences. Sixteen loneliness measures were identified (supplementary materials 1, p2), of which three were selected to inform instrument development. Selection was guided by relevance to the social, emotional, and existential dimensions of loneliness, as well as by the instruments’ conceptual underpinnings, measurement properties, and suitability for nursing home populations (Table 5).

**Table 5:** Characteristics of identified appropriate measurements.

Scale	Items	Initial population	Wording	Dimension
De Jong-Gierveld Loneliness Scale de Jong-Gierveld and Kamphuis, 1985)	11 items; 5-point Likert scale	Dutch adults	Positively and negatively worded	Social and emotional
Shortened De Jong-Gierveld Loneliness Sscale (De Jong-Gierveld and van Tilburg, 2006)	6 items; 5-point Likert scale	Dutch older adults (over 55)	Positively and negatively worded	Social and emotional
Existential Loneliness Questionnaire (Mayers et al., 2002)	22 items; 6 point Likert scale	HIV+ paediatric patients (*N* = 47)	Positively and negatively worded	Existential

The De Jong‑Gierveld Loneliness Scale (DJGLS) emerged as an appropriate indirect measure of social and emotional loneliness in older adults (Bugallo-Carrera et al., 2023). The shortened six‑item DJGLS, widely validated in older populations, was considered suitable because it reduces response burden and demonstrates moderate reliability in individuals with mild-to-moderate dementia (Carbone et al., 2022; Wong et al., 2022). Van Tilburg (2020) suggested applying the seven-item “meaninglessness in life” subscale of the Existential Loneliness Questionnaire (ELQ) to assess existential loneliness among older adults. Although methodological limitations remain, including item ambiguity, response burden, and the possibility that certain aspects of loneliness may remain underrepresented, a combined set of DJGLS items and seven ELQ items, as demonstrated by Schoenmakers and Tromp (2022) appeared to provide the most suitable foundation for a comprehensive assessment of social, emotional, and existential loneliness in nursing home settings.

Subsequently, focus group participants identified themes and observable indicators associated with social, emotional, and existential loneliness, which served as the basis for the initial set of candidate items. Social loneliness was primarily linked to a lack of meaningful social connectedness, emotional loneliness to longing for close relationships, often reflected in withdrawal, repetitive searching behaviours, confusion, and persistent feelings of loss, and existential loneliness to experiences of diminished autonomy, helplessness, and meaninglessness, which were frequently perceived as beyond personal control and therefore associated with feelings of despair. Participants highlighted that measuring loneliness is challenging due to its subjective nature and noted various obstacles. While loneliness could be recognised through verbal statements, emotional expressions, and behavioural cues, peer assessments were considered insufficient and susceptible to caregiver interpretation. Additional barriers included socially desirable responding and fluctuating emotions, particularly among residents with dementia. Suggested strategies to improve assessment involved prolonged observation, multi‑perspective input, the use of residents’ life stories for contextual understanding, informal conversations during daily routines, and visual aids to support residents with cognitive impairments (supplementary materials 1, p9).

### 3.2 Step 2 Content validity

Using a mini-Delphi approach to reach consensus on the candidate items generated in step 1, scientific experts emphasised that assessments should prioritise residents’ own accounts of loneliness, given its subjective nature. Participants noted that loneliness in nursing homes is often expressed through everyday experiences, such as feeling seen, valued, and engaged in reciprocal relationships. This suggested that some aspects of loneliness may be insufficiently reflected in questionnaire items based on the DJGLS and ELQ. Experts further highlighted the multidimensional character of loneliness and recommended including a sufficient number of items across dimensions to ensure comprehensive coverage of the construct and support construct validity (supplementary materials 1, p11).

Following these, the secondary analysis of qualitative studies generated 69 codes related to residents’ experiences of loneliness. While 56 codes corresponded to the social, emotional, and existential items of loneliness as outlined by Van Tilburg (2020), 13 codes did not fit within this framework. These were organised into four institution-specific themes: lack of self-identity, absence of a sense of home, memories of the past, and being alone among others. These themes informed the development of additional items to complement existing loneliness measures (supplementary materials 1, p19). Finally, 18 items adopted from scales as recommended by Van Tilburg (2020), along with 4 items reflecting institutional aspects, were refined into appropriate questions and response options through a co-creation process. This resulted in a final test set of 22 items characterised by simplified wording, a tiered format, and basic response options, thereby enhancing accessibility for residents with cognitive impairment (supplementary materials 1: p22).

### 3.3 Step 3: Pre-testing

Cognitive testing revealed substantial non-completion of the initial questionnaire. In the first round, despite generally higher cognitive functioning among participants, 8 of the 11 respondents did not complete all 22 items (Table 1, “overall score (N)”), with those who discontinued exhibiting an average item-level dropout rate of 26% (supplementary materials 2). To reduce respondents' burden and following the considerations of Wong et al. (2022), items 1, 5, 6, 8, and 9 were removed after the first round, after which five of the remaining items on social and emotional loneliness were aligned with the short 6-item DJGLS.

The overall evaluation of approved items showed that higher Global Deterioration Scale stages were associated with lower average comprehension scores, both for the probes and for overall understanding (Fig. 2). All social and emotional items demonstrated at least sufficient understanding in the first-round sample, except for scale item 6. In contrast, most existential items showed limited comprehension; only items 14 and 18 met the predefined criteria in this sample, characterised by relatively low to Global Deterioration Scale scores. The institutional items were generally sufficiently comprehended, except for item 19. Based on these findings, and after reducing items to lessen the burden, the items were either retained unmodified (2, 3, 4, 7, 10, 11, 14, 18, 21, 22) or rephrased (12, 13, 15, 16, 17, 19, 20).

**Fig. 2:**
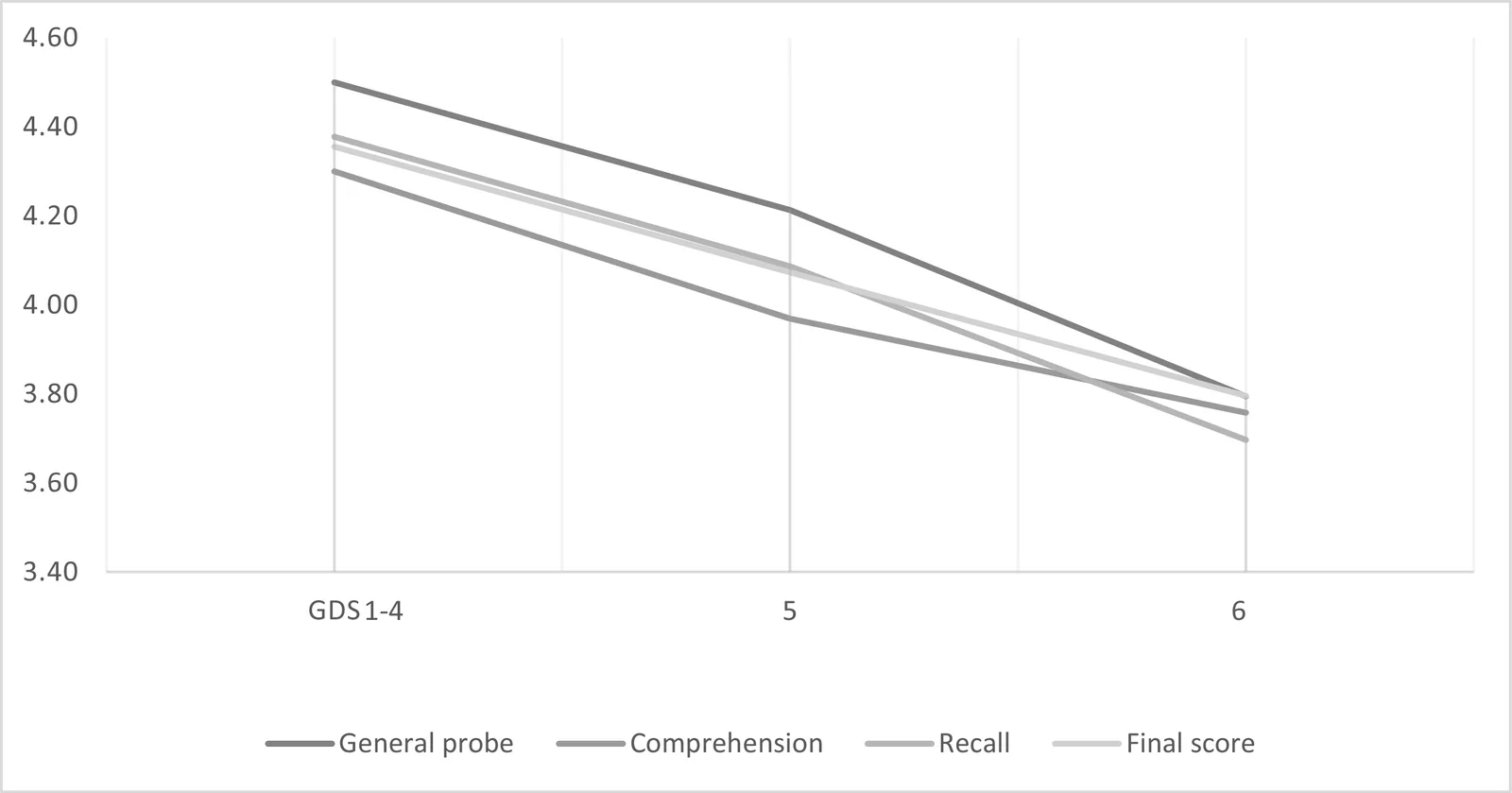
Comprehension related to Global Deterioration Scale (GDS).

In the second-round evaluation of the 10 unmodified items, three social items (2,3,4) and three emotional items (7,10,11) derived from the shortened 6‑item DJGLS showed varied results. Items 3, 4, and 11 continued to show sufficient to adequate understanding, whereas items 2 and 7 were judged to have only marginally sufficient comprehension. When examining the probes for these items, a similar pattern emerged. Probe 1 was assessed as sufficient for both items, indicating that participants generally understood the initial level of questioning. In contrast, probe 2, which introduced expressions central to the thematic content (i.e., “experiencing bad things” and “experiencing emptiness”), scored insufficient comprehension, suggesting that these abstract or affectively charged formulations were less accessible for participants. It was also striking that item 10 (“Do you miss people around you?”) appears to be more sensitive to higher Global Deterioration Scale scores. Fieldnotes indicated that some respondents did not know who the people around them were, whom they would miss. In contrast, both unmodified items, existential loneliness (14 and 18), demonstrated stable comprehension levels across participants. Item 14 (“Do you feel helpless?”) showed consistent understanding, with no observable variation across Global Deterioration Scale stages. Lastly, the second-round evaluation of institutional item 21 (“Do you often feel nostalgic for the past?”) showed a similar sensitivity to higher GDS scores as item 10, where field notes indicated that ’the past’ is a concept that is less understood.

Most existential items already demonstrated insufficient comprehension in the first-round evaluation, even among participants with lower Global Deterioration Scale scores. Despite these limitations, removing the Existential loneliness items was not considered, given their essential role in maintaining the instrument's construct validity. Consequently, five items (12, 13, 15, 16, 17) were rephrased to enhance clarity and semantic simplicity by expressing concrete observations or actions rather than abstract concepts. These modifications were based on field notes and discussions within the research team. Instead, institutional item 19 was revised under the same conditions. Following these adjustments four items (12, 13, 16, and 17) showed substantial improvements in comprehension, despite participants having higher Global Deterioration Scale scores. However, comprehension of existential items 15 and institutional items 19 decreased despite the adjusted phrasing. Moreover, based on interviews and field notes of test round 1, it emerged that item 20, operationalised by the question: “Do you feel attached to this place?”, less effectively inquired about the institutional experiences of a “sense of home”, which is why this question was also modified, despite excellent comprehension scores.

Based on findings from the aforementioned steps and considerations regarding construct validity, and in consensus with the research team, including three senior researchers, a final set of 14 questions was selected, with an average overall sample comprehension score of 4.1 and a range of 3.5–4.4 (Fig. 3). First, for construct validity, we approved 5 items from the shortened 6-item DJGLS (items 2, 3, 4, 7, 11). For item 10, the term “insufficient” was deemed inadequate and was therefore replaced with the more specific emotional item 9: “Do you think your circle of acquaintances is too limited?” This alternative demonstrated sufficient comprehension, even among the 5 participants with Global Deterioration Scale scores above 4.

**Fig. 3:**
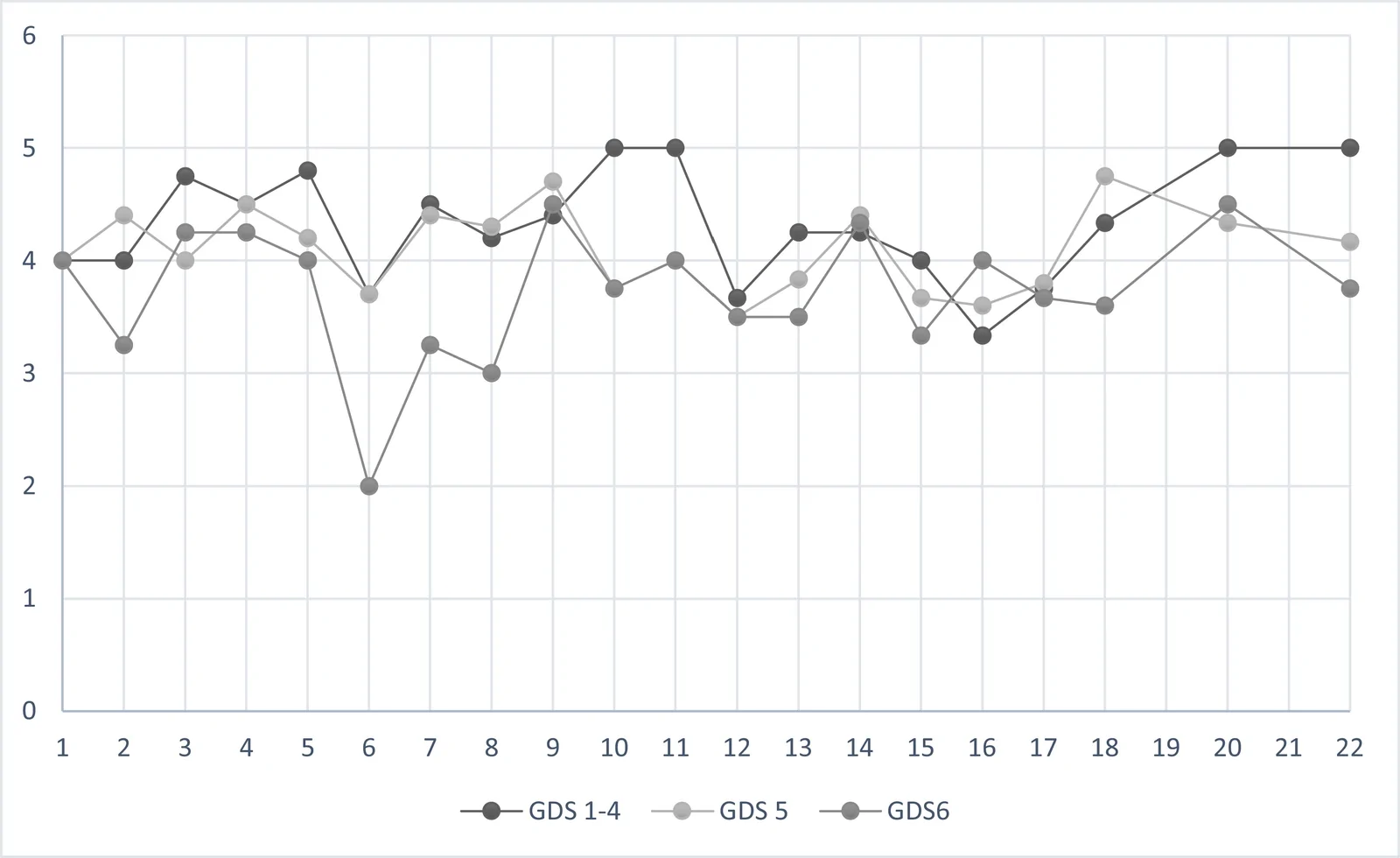
Average item-comprehension score per global deterioration scale stage.

Secondly, the existential items 14 and 18 have been approved for their stability in comprehension and recognition by staff members. The rephrased items 12, 13,16 and 17 are also approved, based on the improvements observed after rephrasing. In contrast, despite rephrasing, item 15 showed insufficient comprehension at the overall second-round sample, and at the individual level and was thus not approved. A reduced validity criterion was applied to the institutional items because they reflect their qualitative origin and are not based on existing valid scales. Due to insufficient comprehension across the sample (item 19) or among the second-round sample (item 21), these items were not approved for the questionnaire (Appendix).

## 4 Discussion

The instrument developed in this study extends existing approaches to loneliness assessment in nursing homes. Unlike widely used measures such as the De Jong Gierveld Loneliness Scale (DJGLS; de Jong-Gierveld & Kamphuis, 1985) and the R-UCLA Loneliness Scale (Russell et al., 1980), which were developed for broader older-adult populations and primarily assess one or two dimensions of loneliness, this instrument was specifically designed for nursing home residents and integrates social, emotional, and existential loneliness along within a single framework. This focus responds to growing recognition that loneliness in nursing homes encompasses experiences related to institutional living that may not be adequately captured by existing measures (Sullivan et al., 2016; Victor, 2012; Zhang et al., 2023). For instance, residents have described loneliness in terms of alienation, loss of control, abandonment, unworthiness, feeling unwelcome, and even confinement (Jansson et al., 2022; Wong et al., 2022; Zhang et al., 2023), highlighting the need for further refinement of loneliness assessment in these settings. A secondary analysis of seven qualitative papers identified several corresponding institutional items that were not adequately captured by existing DJGLS- and ELQ items. Two of which were retained following cognitive interviews: detachment from place (Sundström et al., 2019; Huang et al., 2022) and exclusion, alienation from fellow residents (Barbosa Neves et al., 2019). Within a three-dimensional conceptualisation of loneliness, detachment from place aligns most closely with emotional loneliness (Van Tilburg et al., 2025), whereas experiences of exclusion correspond more closely to existential loneliness (McKenna-Plumley et al., 2024). The combination of established theoretical dimensions and these nursing home-specific experiences may provide a more comprehensive and context-sensitive assessment of loneliness than existing instruments. Content validity was strengthened through expert consultation, residents lived experiences, and iterative cognitive interviewing. Although further psychometric evaluation is required, the instrument has the potential to support more tailored assessment and intervention in nursing practice and to provide a foundation for future validation studies.

A small subgroup of individuals with very advanced cognitive impairments had to be excluded because of communication limitations and ethical considerations. Nevertheless, our findings indicate that many residents with advanced cognitive impairment (including those up to Global Deterioration Scale stage 6) were able to provide meaningful responses to questions about loneliness. Importantly, the ability to do so appeared to depend not only on cognitive status but also on the wording and thematic relevance of individual items. Minor adaptations in phrasing, particularly the inclusion of themes that resonated with residents lived experiences, enhanced comprehension and facilitated meaningful and reliable responses. These findings underscore the importance of tailoring loneliness assessments to the communicative capacities and everyday realities of nursing home residents. However, even with these context-specific adaptations, achieving sufficient comprehension remains challenging among respondents with markedly diminished cognitive abilities. Consistent with prior research (Liu et al., 2019; Kempler and Goral, 2008), higher Global Deterioration Scale scores were, on the sample level, associated with reduced comprehension, reflecting broader impairments in linguistic processing, memory, and executive functioning. However, comprehension varied considerably across items, regardless of Global Deterioration Scale stages. Comprehension is a complex process of decoding, integrating prior knowledge, and constructing a mental interpretation, with natural variations across populations (Ozker et al., 2026). Individual ability varies not only based on cognitive functions but is also influenced by personal factors, such as sensory impairments (e.g., reduced vision and hearing) and educational and cultural differences (Manchha et al., 2025). Furthermore, the context and emotional states, possibly triggered by the question, may influence semantic comprehension (Chwilla, 2022). A significant improvement in comprehension of existential items, however, was achieved by rephrasing items to optimise the semantics and syntax of questions, reduce word count, and avoid the influence of abstract concepts on comprehension. Our findings are not only consistent with Digby et al. (2016) who emphasised that adapted language is essential for improving communication among individuals with diminished cognitive abilities but also highlight its added value for conducting more responsive and meaningful assessments within the broader nursing home population.

Ultimately, achieving a valid and reliable assessment depends on the professionals' level of mastery. Our experiences with cognitive interviewing align with those of Hubbard et al. (2003) who noted that dementia researchers must possess strong verbal and nonverbal communication skills and remain sensitive to how dementia affects memory, decision-making, and emotional expression. Furthermore, Kontos et al. (2017) argued that the body can communicate and express meaning, beyond cognitive and verbal abilities, enabling individuals with advanced cognitive impairments to continue engaging with others through gestures, expressions, and other embodied forms of interaction. During the cognitive interviews, some of our participants were unable to provide a direct verbal answer aligned with the prescribed response options. Instead, they offered associative comments or explanatory narratives. Nonetheless, many of these narratives and contextual cues could still be meaningfully interpreted, thereby guiding the interaction toward a clear and valid answer. This process was facilitated by what O’Shea et al. (2025) described as an intentional stance: empathically attuning to the lived experience of the person with dementia, judiciously interpreting narratives and contextual cues, and guiding the interaction towards a valid response. These findings suggest that accurate loneliness assessment depends not only on appropriate questions and staff experience but also on strong communication skills and a reflective, person-centred approach. Provided these conditions are present, assessments can provide informative and meaningful insights for both residents and staff, reinforcing the importance of including residents with cognitive impairment in loneliness research rather than excluding them by default.

### 4.1 Strengths and weaknesses

A key strength of this study was the rigorous and inclusive assessment of content validity. Item development was informed by existing literature, iterative input from practitioners and academic experts, and the professional experience of the primary researcher, working in a nursing home with people with dementia. Most items were adapted from widely used and validated instruments (DJGLS and ELQ), supporting the content validity. Finally, in line with the guidelines of Boateng et al. (2018), a systematic and reproducible cognitive testing procedure was conducted with a purposive, representative sample, including respondents with and without dementia, to enhance item relevance, comprehensibility, and generalisability. A limitation that should be acknowledged is that item generation was not based on a fully inductive phenomenological exploration of loneliness among nursing home residents. Although extensive cognitive testing and iterative refinement through expert, professional, and lived-experience input enhanced the comprehensibility and relevance of the item pool, this limitation may be particularly pertinent for the existential dimension, given that existential loneliness is strongly shaped by context-specific experiences of ageing, dependency, loss, and institutional living (Bolmsjö et al., 2019; Sundström et al., 2019). Consequently, some existential items may not fully capture how existential loneliness is experienced in long-term care settings, potentially affecting their ecological validity. The recent development of a brief existential loneliness scale (McKenna-Plumley et al., 2024) further illustrates the evolving conceptualization and operationalization of this construct. Future research should therefore examine whether phenomenologically derived items provide additional value in capturing existential loneliness among nursing home residents. Additionally, four existential items needed rephrasing, due to limited comprehension during cognitive testing. Consequently, their approval in the second round was based on a considerably smaller sample. Third, in line with the questionnaire’s primary aim, comprehension scores were evaluated based on the average performance of a representative sample spanning the full spectrum of cognitive deterioration. Items from the shortened DJGLS demonstrated lower comprehensibility among respondents with more advanced impairment, potentially limiting validity in homogeneous samples with severe cognitive decline.

## 5 Conclusion

Using a systematic multimethod approach, this study developed a structured set of items and a practical procedure to assess loneliness in nursing home settings. The most notable finding was that vulnerable residents of nursing home settings with moderate to severe dementia were able to reflect sufficiently on the multi-item scale, which is important because 'loneliness is an intensely personal, subjective feeling’ (Victor et al., 2005). The present study represents an important and necessary early stage in the scale development process. Through a systematic development process involving literature review, expert input, item refinement, and cognitive pre-testing, the instrument underwent key steps to establish content validity and ensure item relevance, comprehensibility, and acceptability. This process resulted in an instrument grounded in established theoretical constructs and validated measures, complemented by nursing home-specific items, thereby providing a strong foundation for subsequent psychometric evaluation and large-scale validation. Further research is recommended before definitive conclusions can be drawn regarding its measurement properties. Consistent with the framework proposed by Boateng et al. (2018), future studies should evaluate reliability and construct validity, including the examination of dimensionality through factor-analytic methods. Additionally, the resulting factor structure should be compared with the underlying theoretical framework to assess structural validity. Finally, cognitive testing indicated that the assessment could facilitate meaningful discussions about residents' experiences of loneliness and unmet needs. However, its effective use depends on trusted staff with expertise in communicating with this population. Embedding the instrument within a structured, reflective administration protocol may therefore maximise its value, both as a research tool and as a means of supporting person-centred care in nursing homes.

## Ethical approval

Ethical approval was obtained from the Erasmus School of Health Policy Ethical Board for all study phases: step 1 (ETH2223–0451) and steps 2 and 3 (ETH2223–0691). The study was conducted in accordance with the principles of the Declaration of Helsinki and complied with the General Data Protection Regulation (GDPR).

## Disclosure statement

The authors reported no potential conflict of interest.

## Funding

This study was funded by members of the Academische Werkplek Ouderenzorg Zeeland (AWOZ): Stichting voor Regionale Zorgverlening Zeeland (SVRZ), Amarijn, and Zorggroep ter Weel. These organisations assisted in the recruitment of participants for focus groups and cognitive interviews. The funders had no role in data analysis, data interpretation, the drafting of the manuscript, or the decision to submit the paper for publication.

## CRediT authorship contribution statement

**Dick van de Kolk:** Writing – original draft, Validation, Resources, Project administration, Methodology, Investigation, Formal analysis, Data curation, Conceptualization. **Robbert Huijsman:** Writing – review & editing, Supervision, Funding acquisition, Conceptualization. **Dirk Frans de Korne:** Supervision, Conceptualization. **Gerda Andringa:** Writing – review & editing, Supervision, Methodology, Conceptualization.

## Declaration of competing interest

The authors declare that they have no known competing financial interests or personal relationships that could have appeared to influence the work reported in this paper.

## Figures and Tables

**Table:** 

Item	Dimensie	Vraag	Respons		
			Nee	Min of meer	Ja
1	Sociaal	*Als u narigheid overkomt, zijn er dan genoeg mensen die u steunen?*	2	1	0
2		*Zijn er veel mensen die u volledig kunt vertrouwen?*	2	1	0
3		*Zijn er genoeg mensen met wie u een goede band heeft?*	2	1	0
4	Emotioneel	*Ervaart u een leegte om u heen?*	0	1	2
5		*Vindt u uw kring van kennissen te klein?*	0	1	2
6		*Voelt u zich vaak in de steek gelaten?*	0	1	2
7		*Voelt u zich hier thuis?*	2	1	0
8	Existentieel	*Voelt u zich buitengesloten?*	0	1	2
9		*Kijkt u terug op een gelukkig leven?*	2	1	0
10		*Geeft het leven u voldoening?*	2	1	0
11		*Voelt u zich hulpeloos?*	0	1	2
12		*Denkt u vaak “zo hoeft het leven niet meer voor mij”?*	0	1	2
13		*Kunt u de dingen doen die u graag wilt doen?*	2	1	0
14		*Is uw leven zinloos?*	0	1	2

**Table:** 

Item	Dimension	Question	Response		
			No	More or less	Yes
1	Social	*If you experience bad things, are there enough people who support you?*	2	1	0
2		*Are there many people you can completely trust?*	2	1	0
3		*Are there enough people with whom you have a close relationship?*	2	1	0
4	Emotional	*Do you experience a sense of emptiness around you?*	0	1	2
5		*Do you think your circle of acquaintances is too limited?*	0	1	2
6		*Do you often feel left behind?*	0	1	2
7		*Do you feel at home here?*	2	1	0
8	Existential	*Do you feel excluded?*	0	1	2
9		*Do you look back on a happy life?*	2	1	0
10		*Does life give you fulfilment?*	2	1	0
11		*Do you feel helpless?*	0	1	2
12		*Do you often think “life doesn't have to be like this for me anymore”?*	0	1	2
13		*Are you still able to do the things you enjoy doing?*	2	1	0
14		*Does life have little meaning for you?*	0	1	2

## Appendix: Multidimensional assessment scale for loneliness in nursing homes

Multidimensional assessment scale for loneliness in nursing homes (Original Dutch version)

Multidimensional assessment scale for loneliness in nursing homes (Englisch translation)
