# Supplementary material for: The development and preliminary validation of a multidimensional assessment scale for loneliness in nursing homes
Source: Int J Nurs Stud Adv. 2026 Sep 24;11:100685. doi: 10.1016/j.ijnsa.2026.100685 (PMC13653931; doi:10.1016/j.ijnsa.2026.100685)
Supplement: Supplementary file 1 [file mmc1.docx]

**Supplementary Materials**

**Title of manuscript:**
The development of a multidimensional assessment scale for loneliness in nursing homes

**Authors:**
<..>

**Journal:**
International Journal of Nursing Studies Advances

**Corresponding author:**
<…>

**Contents of this supplementary file**

Section 1 Item generation	2

1.1.	Result of the narrative literature review	2

1.2.	Protocol focusgroups with staff (translated from original Dutch version)	4

1.3	Analysis focusgroups with staff; codebook	8

1.4	Summary of results focus groups with staff (17 particpants)	9

Section 2 Content validity	11

2.1	Consensus report of Mini -Delphi	11

2.1.1	Particpants (n=5)	11

2.1.2	Delphi consensus findings	11

2.1.3	Delphi round 1: Results of the questionnaire	12

2.1.3.1	Loneliness as a (total) concept, scale items	12

2.1.3.2. Theme 1 (Presence) of meaningful relationships	12

2.1.3.3. Theme 2 : Longing for intimate relationships	13

2.1.3.4. Theme 3 and 4: Helplessness and Meaninglessness	14

2.1.3.5	Assessing by proxy’s	15

2.1.3.6. Procedure: Response	15

2.1.3.7. Timeframe of assessments	16

2.1.4.	Delphi Round 2 Focus Group	17

2.1.4.1 Assessing loneliness among residents of nursing home settings	17

2.1.4.2. Considerations regarding operationalization and definitions	17

2.1.4.3. Considerations regarding the distinction in dimensions	17

2.1.4.4. Considerations for Scale Item and Content	18

2.1.4.5. After the development of a scale	18

2.1.4.6. Additional Suggestions	18

2.2.	Institutional aspects a result of secondary analysis.	19

2.3	Evolution wording in expertgroups	22

Section 3 Pre-testing	24

3.1. Protocol Cognitive interviewing	24

# Step 1 Item generation

## 1.1. Result of the narrative literature review

| **Scale** | **Reference** |
| --- | --- |
| 10-UCLA | Knight et al., 1988 |
| 3-UCLA | Hughes et al., 2004 |
| 6-JGS | De Jong-Gierveld & Van Tilburg, 2006 |
| Belcher Extended Loneliness Scale | Belcher 1973 |
| CLS | Asher, Hymel & Renshaw, 1987 |
| The Jong-Gierveld scale | De Jong-Gierveld & Kamphuis 1985 |
| Differential Loneliness Scale | Schmidt & Sermat, 1983 |
| Existential loneliness questionnaire | Mayers & Svartberg, 2002 |
| HAIRSPRAY | Marcoen, Goossens & Caes, 1987 |
| Loneliness Rating Scale (LRS) | Scalise 1984 |
| Peer Network and Dyadic Loneliness Scale | Hoza, Bukoski & Beery, 2000 |
| Revised UCLA-LS (R-UCLA) | Russell, 1980 |
| RPLQ | Thompson, 1989 |
| Social and Emotional Loneliness Scale for Adults (SELSA) | DiTomasso & Spinner, 1993 |
| ULS-8 | Hays, DiMatteo & Robin, 1987 |
| University of California Los Angeles - Loneliness Scale (UCLA-LS) | Russell, Peplau & Ferguson 1978 |

**References:**

Asher SR, Hymel S, Renshaw PD. Loneliness in children. Child Dev. 1984; 55(4):1456-1464.

Belcher M. The measurement of loneliness: a validation study of the Belcher Extended Loneliness Scale (BELS) [doctoral dissertation]. London: University of London; 1973.

de Jong Gierveld J, Kamphuis F. The development of a Rasch-type loneliness scale. Appl Psychol Meas. 1985; 9(3):289-299.

de Jong Gierveld J, van Tilburg T. A 6-item scale for overall, emotional and social loneliness: confirmatory tests on survey data. Res Aging. 2006; 28(5):582-598.

DiTommaso E, Spinner B. The development and initial validation of the Social and Emotional Loneliness Scale for Adults (SELSA). Pers Individ Dif. 1993; 14(1):127-134.

Hays RD, DiMatteo MR. A short-form measure of loneliness. J Pers Assess. 1987; 51(1):69-81.

Hoza B, Bukowski WM, Beery S. Assessing peer network and dyadic loneliness. J Clin Child Adolesc Psychol. 2000; 29(1):119-128.

Hughes CP, Berg L, Danziger W, Coben LA, Martin RL. A new clinical scale for the staging of dementia. Br J Psychiatry. 1982; 140(6):566-572.

Knight RG, Chisholm BJ, Marsh NV, Godfrey HP. Some normative, reliability, and factor analytic data for the revised UCLA Loneliness Scale. J Clin Psychol. 1988; 44(2):203-206.

Mayers AM, Khoo ST, Svartberg M. The Existential Loneliness Questionnaire: background, development, and preliminary findings. J Clin Psychol. 2002; 58(9):1183-1193.

Marcoen A, Goossens L, Caes P. Loneliness in pre- through late adolescence: exploring the contributions of a multidimensional approach. J Youth Adolesc. 1987; 16(6):561-577.

Scalise JJ, Ginter EJ, Gerstein LH. Multidimensional Loneliness Measure: the Loneliness Rating Scale (LRS). J Pers Assess. 1984; 48(5):525-530.

Schmidt N, Sermat V. Measuring loneliness in different relationships. J Pers Soc Psychol. 1983; 44(5):1038-1047.

Thomson L. The development of the relational provision loneliness questionnaire for children [dissertation]. Waterloo (ON): University of Waterloo; 1989.

Russell D, Peplau LA, Ferguson ML. Developing a measure of loneliness. J Pers Assess. 1978; 42(3):290-294.

Russell D, Peplau LA, Cutrona CE. The revised UCLA Loneliness Scale: concurrent and discriminant validity evidence. J Pers Soc Psychol. 1980; 39(3):472-480.

## 1.2. Protocol focusgroups with staff (translated from original Dutch version)

**Date:** 23-02-2023 (focusgroup 1) and 08-03-2023 (focusgroup 2)

*Research into the identification of loneliness in the nursing home setting. The research is part of the PhD research on loneliness in the nursing home setting. The aim of the focus groups is to collect knowledge and experiences from healthcare professionals from the care practice of the nursing home about identifying loneliness.*

|  | **Introduction** | | | | | |
| --- | --- | --- | --- | --- | --- | --- |
|  | **15.00** | | ***Introduction*** | | *Welcome everyone. Thank you for participating in the research into identifying loneliness! My name is <.. >, and I am a researcher at <.. > , I am doing this part of the research together with < research assistant> ( "I study psychology and economics at <.. >”)* | |
|  |  | | ***Purpose of the study*** | | *Since this is a scientific study, a more formal part will now follow. You have received information and a declaration of consent about the research and the group discussion. This information in brief:*    *The aim of the research is to develop a method with which loneliness can be identified in the nursing home setting (explanation). We want to draw this up using existing methods for measuring loneliness. In addition, we need your knowledge and experience to draw up a method to identify loneliness. Healthcare professionals like you are the ones who will ultimately have the most to do with this method. In this focus group, we will ask you to assess the propositions of an existing method for use in the nursing home based on your experiences. To this end, we are going to ask a number of questions about the content of the statements, and how they can best be offered to the residents. It is important to know that there are no right or wrong answers.* | |
|  |  | | ***Informed consent*** | | *The information collected will be treated confidentially. The invitation letter explains the data storage and processing and how we handle your personal data. Does anyone have any questions or comments about that?*  *The declarations of consent can now be handed in and if not yet signed, there is now the opportunity to sign.* | |
|  |  | | ***Program*** | | *The program of the next 2 hours is built up in parts. Part 1 is an introduction to loneliness and the dimensions, forms of loneliness. Parts 2, 3, and 4 are devoted to statements with which loneliness can be signaled. In these parts of the program, you are asked to give your opinion via a vote on the website WOOCLAP.*  *Instructions on how to connect to <Wi-Fi network> and the web page can be found on the paper that is handed out to you.*    *In the last part, questions are asked about how we can best offer the questionnaire*  *I lead the conversation and < research assistant> take notes, monitor the time schedule and adjust the conversation if necessary.*  *Of course, you can always choose to leave the conversation if you feel uncomfortable with a topic. You can also always leave the room to go to the toilet, for example. No interim break is planned.* | |
|  |  | | ***Data*** | | *The conversation will be recorded, both image and sound. This is necessary for a good transcription, in which the literal text of the research is written out. We record images to check who is speaking, this is not always easy to distinguish on the audio recording. After transcription, the recordings are deleted. In the transcription, the names are replaced by participant numbers, so that anonymity is guaranteed. By means of analysis of the text, information is obtained about the usability of items or statements for the measurement method and from whom and how we can obtain this information. At the end of this analysis, a summary of the results of the survey will be shared by email with the question whether you recognize these results. Please note that there may be several months between the survey and the mailing.*  *Are there any questions about the program?* | |
|  |  | | ***Recording*** | | *The call is now recorded, audio (recorder) and image (teams).* | |
|  |  | | ***Naming tasks*** | | *We, <.. name researchers.. > are researchers within the sub-study on the identification of loneliness, and we will conduct the group interview with you. Mention [date] and focus group employees person-centered care and support / housing and welfare* | |
|  | **15.15** | | ***Proposals***    - Department  - Function  - How long have you been working in the position | | *Can you all briefly introduce yourself?*    *Sub-questions:*  *Which organization and department(s) do you work for (in terms of: "residential department for psychogeriatrics, or somatics or something similar)*   - *What position do you work in?* - *How long have you been working with these residents in this position?* | |
|  | **15:20** | | 1. ***Loneliness and identifying and discussing***  - example loneliness | | *The subject of Loneliness in the nursing home setting, identifying and discussing it has been > topic at < name research institute in the development of an e-learning for employees. The next item at Omroep Zeeland was made at the time about this theme < start film fragment>*  *You have seen a fragment in which a caregiver was involved and a resident who has to deal with feelings of loneliness.*  *Everyone feels lonely sometimes, how do you notice loneliness in others or in yourself?*    *Loneliness can have a social origin, a lack of the amount of contacts, an emotional origin (a lack of a deep connection) with one or more people, or an existential origin, a feeling of "not mattering anymore"*  *How would you rate the loneliness of the resident?  What is this judgment based on?* | |
|  | **15:30** | | ***Introduction usability of the items by dimension.***    Instruction Ranking Items by Dimension | | *As mentioned, loneliness is divided into social, emotional and existential loneliness, based on this classification we will now discuss loneliness. We do this with the help of statements that can be used to identify this form of loneliness.*  *When you enter the code into your phone, the statements are presented on the screen.  You choose the statement that you think is best to identify, so that is easy to observe, that means the same thing to everyone, and about which a more or less reliable statement can be made. We conduct the conversation on the basis of the order of the statements that arises on the screen.* | |
|  | | ***Social loneliness***  Items:   - Ranking - Demarcation - Unambiguity Interpretation - Reliable determination | | *Social loneliness is the lack of social connections and contact. For example, missing friends. The actual social contact is less than the social need.*    *The 5 statements about social loneliness are*:   - *In daily problems, the resident does not experience any support from his/her environment.* - *In case of trouble, the resident cannot count on people from his/her environment.* - *The resident has few people around him/her that he/she trusts.* - *The resident does not feel connected to people in his or her immediate environment* - *The resident lacks friends with whom he/she can express himself directly and on whom he can count.*   *Now choose from one of these statements (5 JGS items), via your phone. Choose the statement that demonstrates best measurable*    *What makes that statement .. is the best to determine ? Is there another statement in which this more difficult aspect is different?*  *What differences in the image of the proposition is... .*  *What behavior can be seen/noticed by the resident......* | |  |
|  | **15:50** | | ***Emotional loneliness***  Items:   - Ranking - Demarcation - Unambiguity Interpretation - Reliable determination | | *Emotional loneliness is a need for an intimate connection or relationship. This can be a partner or a best friend, for example*    *The 6 statements about emotional loneliness are:*   - *The resident feels a deep longing for a person who can be a true friend.* - *The resident feels empty.* - *The resident misses cosiness in his/her living environment.* - *The resident is not satisfied with the number of acquaintances he/she has.* - *The resident misses people around him.* - *The resident feels abandoned.*     *Now choose from one of these statements (6 JGS items), via your phone. Choose the statement that demonstrates best measurable.*    *What makes that statement .. is the best to determine ? Is there another statement in which this more difficult aspect is different?*  *What differences in the image of the proposition is... .*  *What behavior can be seen / notice that the resident....* | |
|  | **16:10** | | ***Existential loneliness***                            Items:   - Ranking - Demarcation - Unambiguity Interpretation - Reliable determination | | *Existential loneliness is the lack of meaning to life, or the feeling of not being connected to others and the world. Feeling misunderstood. Is separate from contact with (important) others.*    *The 7 statements about emotional loneliness are:*  *The resident is unhappy with the life he/she has led.*   - *The resident experiences that his/her life has no purpose, meaning.* - *The resident feels helpless.* - *The resident feels powerless in the face of the world around him. The resident must experience his/her situation as if he/she is no longer alive.* - *The resident sees many possibilities in his/her living environment.* - *The resident experiences life as meaningless.*     *Now choose from one of these racks (7 ELQ items), via your phone.  Choose the statement that demonstrates best measurable*    *What makes that statement .. is the best to determine ?*  *Is there another statement in which this more difficult aspect is different?*  *What differences in the image of the proposition is... .*  *What behavior can be seen/noticed by the resident......* | |
|  | **16:30** | | ***Shape of the shell***       - Who to ask - How to question - Tools/ tools - Reliability - Direct question | | *You have just given your experiences and views on the usefulness of the theses.*    *We also want to know how we can best identify these items*:   - *Who can we best question about these statements?* - *If we want to question residents, do you have any suggestions for how we can do this? (think for example of all residents, including people with a form of dementia, can they be questioned?)* - *Do you have any suggestions for any tools we could use to get a reliable answer to the statements? Consider, for example, a traffic light method.* - *Are there any other things we can take into account?*   *Finally.*  *There are several methods to determine loneliness. The previous statements avoid the word "loneliness".  People may give a less reliable answer to the direct question "do you suffer from loneliness?"*    *What is your experience about the reliability of the answers if you were to ask your residents directly about loneliness?* | |
|  | 16.55 | | ***Conclusion*** | | *We thank you very much for your participation and contribution to this group discussion. We will now end the recording.*    *If you have any questions or comments, you can still speak to us afterwards. You can also exchange ideas with us at a later time by e-mail, see the e-mail address on the information letter.* | |
|  | *Note*. Both focus groups used this protocol, with only minor changes related to the different functions of the participants. | | | | | |

## 1.3 Analysis focusgroups with staff; codebook

| **Number** | **Category** | **Theme** | **Code** |
| --- | --- | --- | --- |
| 1.1 | Loneliness | Recognizing emotions | Explicit expression |
| 1.2 | Loneliness | Recognizing emotions | Implicit expression |
| 2.1 | Social loneliness | Meaningful relationships | Origin |
| 2.2 | Social loneliness | Meaningful relationships | Type |
| 2.3 | Social loneliness | Meaningful relationships | Quality |
| 2.4 | Social loneliness | Meaningful relationships | Subjective experience |
| 2.5 | Social loneliness | Meaningful relationships | Recognition of identity |
| 2.6 | Social loneliness | Setting | Environment |
| 2.7 | Social loneliness | Setting | Impairments |
| 2.8 | Social loneliness | Setting | Familiarity |
| 2.9 | Social loneliness | Setting | Participation |
| 3.1 | Emotional loneliness | Longing for intimate connections | Physical separation |
| 3.2 | Emotional loneliness | Longing for intimate connections | Searching behaviours |
| 3.3 | Emotional loneliness | Longing for intimate connections | Lingering emotions |
| 3.4 | Emotional loneliness | Longing for intimate connections | Repetition |
| 3.5 | Emotional loneliness | Longing for intimate connections | Confusion |
| 3.6 | Emotional loneliness | Longing for intimate connections | Inability to express |
| 3.7 | Emotional loneliness | Longing for intimate connections | Staff identity |
| 3.8 | Emotional loneliness |  | Social vs. emotional |
| 4.1 | Existential loneliness | Loss of autonomy | Dependence |
| 4.2 | Existential loneliness | Loss of autonomy | Fear |
| 4.3 | Existential loneliness | Loss of autonomy | Realisation |
| 4.4 | Existential loneliness | Helplessness | Self |
| 4.5 | Existential loneliness | Helplessness | Others |
| 4.6 | Existential loneliness | Helplessness | Realisation |
| 4.7 | Existential loneliness | Meaninglessness | Apathy |
| 4.8 | Existential loneliness | Meaninglessness | Feeling like one no longer lives |
| 4.9 | Existential loneliness | Meaninglessness | Loss |
| 4.10 | Existential loneliness | Meaninglessness | Not looking for meaning |
| 4.11 | Existential loneliness | Meaninglessness | Seeing no opportunities |
| 4.12 | Existential loneliness | Meaninglessness | Expressions of despair |
| 4.13 | Existential loneliness | Meaninglessness | Realisation |
| 4.14 | Existential loneliness | Meaninglessness | Comprehension |
| 4.15 | Existential loneliness | Meaninglessness | Inability to improve |
| 5.1 | Practical considerations | Signalling | Observation |
| 5.2 | Practical considerations | Signalling | Verification |
| 5.3 | Practical considerations | Signalling | Discrepancy |
| 5.4 | Practical considerations | Measurability | Changeability |
| 5.5 | Practical considerations | Measurability | Social desirability |
| 5.6 | Practical considerations | Measurability | Inferences |
| 5.7 | Practical considerations | Measurability | Multiple perspectives |
| 5.8 | Practical considerations | Measurability | Conversation |
| 5.9 | Practical considerations | Measurability | Role of lifeline |
| 5.10 | Practical considerations | Measurability | Role of others |

## 1.4 Summary of results focus groups with staff (17 particpants)

The results have been categorized into two groups: themes by which loneliness can be identified in the nursing home setting, and the measurement of loneliness in practice.

**1. Characteristics and Dimensions of Loneliness in Nursing Home Settings.**.

The findings indicate that apathy and feelings of emptiness are general indicators of loneliness. Loneliness can first be recognized through explicit statements by residents, such as, “I am just alone,” or “I feel like I am no longer living.” More often, however, this sense of emptiness is revealed through indirect cues or implicit expressions. Residents may manifest these feelings through body language or tone of voice. Certain behaviors, such as social isolation, denial, searching behavior, or misunderstood conduct, can also serve as signals of loneliness.

During the focus group discussions, three dimensions of loneliness were examined, and statements were presented that may aid in identifying each specific dimension of loneliness.

a. Social loneliness (a lack of connection with those around the individual)

The discussions revealed that the absence of interpersonal connections may indicate social loneliness. In the nursing home context, “others” may refer to family, friends, staff, or fellow residents. Various types of connections exist, including friendly or functional relationships. Dependence and trust play significant roles in the relationships between residents and care staff. Assessing the quality of these connections from the resident’s perspective is challenging, as it remains a subjective experience.

Certain features of the nursing home environment complicate the establishment of relationships. For instance, residents cannot choose their housemates, and some individuals on a ward may set the tone for the overall atmosphere. Furthermore, physical and/or cognitive impairments can hinder residents’ ability to form or maintain connections. Finally, familiarity and predictability within the environment may be prerequisites for some residents to engage in relationships.

b. Emotional loneliness (the absence of an emotionally close bond with one or more individuals)

Emotional loneliness may manifest as a longing for an intimate relationship or bond. This can be explicitly stated, or expressed implicitly. Indirect indicators include physical separation from a partner, searching behavior, repetition, and incomprehension. For individuals with dementia, it can be especially difficult to understand that the person they miss is no longer accessible. Additionally, lingering emotions after a loved one’s departure can also indicate a close bond between the resident and that individual.

c. Existential loneliness: a sense of “no longer mattering” or lacking significance.

Three themes emerged regarding existential loneliness: loss of autonomy, feelings of powerlessness, and a sense of meaninglessness. Loss of autonomy may be demonstrated by fear or dependence on others, which is closely related to experiencing powerlessness and an inability to care for oneself or others. In some cases, residents experience meaninglessness, which may be expressed through apathy, experiences of loss, expressions of despair, or no longer seeking meaning in life. A key feature of existential loneliness is that, in contrast to social and emotional loneliness, residents often feel unable to change their situation.

**2. Measuring Loneliness in Nursing Homes**

Two main topics emerged regarding the assessment of loneliness among nursing home residents.

First, loneliness can be identified through the observations of care staff. It is important that staff verify whether their observations are accurate and whether the resident genuinely experiences loneliness.

The second topic concerns specific suggestions for measuring loneliness. Several factors complicate the assessment process. For example, it was noted that responses to existing scale items are often more a reflection or interpretation by the care provider than the resident’s own experience. It is also important to be aware of socially desirable responses and the variability of emotions, especially in residents with dementia. For these reasons, it was suggested that care staff should observe residents over a longer period. It also emerged that assessing loneliness is most effective when undertaken from multiple perspectives and by multiple people. A resident’s life history can provide important context for better understanding their experience of loneliness. Conversations about loneliness are best conducted in informal settings, such as during household chores or while walking. For residents with dementia, the use of visual aids in conversations may be beneficial. Care staff play a crucial role in measuring loneliness, and discussing residents’ loneliness among staff can improve identification.

# Step 2 Content validity

## 2.1 Consensus report of Mini -Delphi

### 2.1.1 Particpants (n=5)

Resp. 1. Expert in (care ethics) meaning

Resp. 2. Expert in Loneliness

Resp. 3. Expert in care ethics (meaning) and dementia care

Resp. 4. Expert in care ethics (meaning) and dementia care

Resp. 5. Expert in loneliness

### 2.1.2 Delphi consensus findings

1. In nursing home settings, the experience of loneliness seems to be more difficult to assess than in a general population, due to specific characteristics of the population (dependency, loss, physical and cognitive limitations) and specific characteristics of the settings (often involuntary or forced by circumstances living with others in groups, dependence on a care system).
2. Loneliness is locked into the everyday experiences in the residents' specific environment (such as "feeling seen and heard", "mattering", and "reciprocity within the relationship") These everyday experiences may better connect residents of nursing home settings to the selected items of the existing scales. It is therefore suggested to further explore the experience of loneliness of nursing home residents in their daily lives in the settings.
3. If a rating scale for loneliness in nursing home settings is nevertheless based on items from the existing scales, item reduction is not recommended, partly because it is doubtful whether the items on their own, or in a selection, sufficiently describe the full breadth of the loneliness experience in this population.
4. If item reduction is nevertheless considered (e.g. because longer scales are more difficult to administer in people with dementia), the selection can best be based on the results of a factor analysis of data from research in comparable populations. It may also be considered to base the scale on the 6-item JGS (for assessing social and emotional loneliness).
5. The involvement of formal caregivers is conditional in the development and validation of a scale to be able to assess (dimensions) of loneliness in the nursing home setting. Further research, in relation to the circumstances and the time of measurement, is needed to properly assess the pronunciation of the residents with dementia.
6. The interrelationship between the dimensions must also be taken into account, This relationship complicates measuring; Statements about connecting items (such as "feeling empty") can refer to multiple dimensions of loneliness.
7. The assessment should primarily take place with the resident (by means of a form of questioning) because of the subjective experience of loneliness. An assessment of residents, collected from care workers or informal caregivers, can be useful, but can also give distortion.

### 2.1.3 Delphi round 1: Results of the questionnaire

#### 2.1.3.1 Loneliness as a (total) concept, scale items

**1a. Is a direct question "do you sometimes feel lonely" a reliable question to assess loneliness in people in the nursing home, including people with dementia?**

A direct question is not reliable enough to assess loneliness among residents of vph settings. It may be considered to ask this question at the end of the assessment for verification.

**1b. Four themes (related to scale items) from a selection of 18 scale items were considered by care workers to be the most useful (measurable) for measuring dimensions of loneliness. Assess whether an assessment of the above 4 items can be used to make a good statement about the degree of loneliness (total) that a resident of the nursing home experiences.**

- **Theme1 : (*Presence) of meaningful relationships***
- **Theme 2: *Longing for intimate relationships***
- **Theme 3: *Helplessness***
- **Theme 4: *Meaninglessness***

Submitting items related to these 4 themes (from the analysis of the focus group discussions with employees) does not seem to be sufficient for a good assessment of loneliness, because (1) items (such as "feeling seen and heard") are missed, (2) items do not correspond to the definition of loneliness (a lack of..., instead of longing for...") and (3) there are comments in terms of content about the items of existential loneliness mentioned (why should Helplessness and meaninglessness are loneliness? ).

*Note: Limited reliability due to comments from participants on the conceptualization.*

**1c. Which additional themes, in addition to the 4 aforementioned themes, should in your opinion be at least assessed in order to arrive at a good statement about loneliness (several choices possible). ^[1]^**

None of the suggested items are clearly apparent as complementary to the 4 items presented. "Being part of a larger whole", being seen as a human being, connecting with others" and "acknowledging loss" are items that are more in line with loneliness in the nursing home setting. These items are mentioned by multiple experts.

#### 2.1.3.2. Theme 1 (Presence) of meaningful relationships

**2a. Assess whether an assessment of theme 1 can be used to make a good statement about the social loneliness experienced by a resident of the nursing home.**

An assessment of *(the presence of) meaningful relationships* is considered sufficient to make a good statement about social loneliness. However, a few respondents interpreted the question count as a proposal for an alternative conceptualization, limiting the validity of this result.

**2b. This theme is most similar to the item (statement) of the van De Jong Gierveld loneliness scale (DJG): "*There are plenty of people with whom I feel closely connected".***

**Is this statement sufficiently understandable for nursing home residents, including people with dementia?**

In addition to question 2A, it is suggested that the items of the abbreviated JGS should be taken as a basis for an assessment and that further research be done into how these items should be administered. Suggestions are made with regard to illustration of the question with gestures and more concrete language that is in line with the lived experience ("are there people with whom you have a nice contact, who you like to be around and who you can turn to with questions?").

**2c. How can the statement best be assessed/questioned by the resident?**

In order to arrive at an objective assessment, specifically in people with a cognitive disability, further research is recommended. It is suggested that an assessment can best be entered into through the conversation, using non-vebal communication and supported by images

#### 2.1.3.3. Theme 2 : Longing for intimate relationships

**3a. Assess whether an assessment of theme 2 can be used to make a good statement about the emotional loneliness experienced by a resident of the nursing home.**

To arrive at a good statement about the dimension of emotional loneliness, a statement about the item "*the desire for intimate relationships"* seems to be insufficient, additional items such as "feeling seen and heard" are suggested by the respondents.

In addition, a few respondents interpreted the question count as a proposal for an alternative conceptualization, which limits the validity of this result.

**3b. This theme is most similar to the item (statement) of De Jong Gierveld loneliness scale (DJG): "*I miss a really good friend*".**

**Is this statement sufficiently understandable for nursing home residents, including people with dementia?**

In addition to question 2A, it is suggested that the items of the abbreviated JGS should be taken as a basis for an assessment and that further research be done into how these items should be administered. Suggestions are made with regard to clarification of concepts and accents of the question.

**3c. How can the statement best be assessed/questioned by the resident?**

Equal to 2c.

#### 2.1.3.4. Theme 3 and 4 : Helplessness and Meaninglessness

##### **4a. Assess whether an assessment of themes 3 and 4 can be used to make a good statement about the existential loneliness experienced by a resident of the nursing home.**

There are hesitations, especially with the concept of helplessness in relation to existential loneliness, existential loneliness can mainly be assessed on the basis of the items "meaninglessness" and "not feeling seen and heard"

Some respondents interpreted the question count as suggesting an alternative conceptualization, limiting the validity of these results.

**4b. Theme 3 is most similar to the item (statement) of the Existential Loneliness Scale (ELQ): "*I feel helpless*".**

**Is this statement sufficiently understandable for nursing home residents, including people with dementia?**

More research seems to be needed into adequate items and their wording in existential loneliness. There are comments on helplessness in relation to loneliness. With further questioning, more meaning (such as arising from "a feeling of desperation") could be given to this item.

**4c. How can the statement best be assessed/questioned by the resident?**

Equal to 2c.

**4d. Theme 4 most closely corresponds to the item (statement) of the JGS loneliness scale: "*Life has little meaning."***

**Is this statement sufficiently understandable for nursing home residents, including people with dementia?**

This item also requires a sharper formulation and room for verification (e.g. "desired answer based on religious belief"), as well as the aforementioned comments in relation to the concept of loneliness.

Some respondents interpreted the question count as meaning that an alternative conceptualisation was proposed, which limits the validity of it.

**4e. How can the statement best be assessed/questioned by the resident?**

Equal to 2c .

#### 2.1.3.5 Assessing by proxy’s

 **5a. Which assessors should be additionally involved in a proper assessment of the items?**

There seems to be no agreement between the experts. On the one hand, involving several people, such as informal caregivers or care workers, can lead to a better assessment. On the other hand, the purpose of doing this is questioned, pitfalls are identified that stand in the way of an objective assessment: (such as wanting to "hide one's own suffering from the children") or it is doubted whether these people (around the resident) are able to form a good picture of the loneliness of the residents.

**5b. To what extent is there an unambiguous significance for the assessors involved in question 5a?**

**5c. Is the item sufficiently specific for the person concerned of question 5a?**

These questions were insufficiently understood, or it was concluded that they are too premature for this phase of the study.

#### 2.1.3.6. Procedure: Response

**6. When assessing the items in residents, what is optimal response scale/possibility (taking into account cognitive and communicative impairments)?**

There is no consensus among the experts on this question. With the choices yes / no, possibly with a middle answer (neutral), experience has been gained with the elderly, in which the limitation of options worked out well. Grades 0-10 provide more nuance, positive experiences have been gained in palliative practice, also with people with dementia. It is also suggested to follow the proven response scale of the original questionnaire (JGS).

#### 2.1.3.7. Timeframe of assessments

**7. Assessments momentary or multiple assessments (average)**

There is a limited consensus among the experts that an assessment is best done in the administration. Specific disadvantages of multiple decreases over time (test-retest) are "the situation may change", or in the case of a decrease in a previous period " loneliness may be fleeting. Multiple questions can be asked in 1 conversation, which can contribute to feeling "seen" and "heard". 1 respondent suggested integrating the samples into a broad cycle of conversations.

### 2.1.4. Delphi Round 2 Focus Group

**Date: 07-06-2023**

**Participants:** *R1,R2,R3,R4*

#### 2.1.4.1 Assessing loneliness among residents of nursing home settings.

- The disease (dementia) by definition brings with it a feeling of loneliness, how to distinguish between this fundamental feeling and "factors"? To then make a statement about factors (such as a care concept) is problematic *("a tricky exercise")*
- Measuring in dementia: is more and more of a snapshot during the course, The experience goes up and down "lived experience" what is the value of measuring (and what does that say about differences in care concepts or circumstances)
- People with dementia are less able to express themselves, which requires more skills among employees to identify this.
- In the nursing home, loneliness is mainly locked up in concrete experiences: standard measuring instruments do not provide for this. As an example: Attention to the small daily things in that shrunken world is very important. This is rarely reflected in working with the 3 dimensions or a translation has to be made.
- An essential item of loneliness in a home setting is: "feeling seen and heard", "making a difference". *This can also be done, for example, in a short wave contact".* Reciprocity ("interaction") in a relationship is also important. These items do not appear explicitly in items of the current scales.
- It is precisely these items that are often missed by care workers, so that will also come up less when you ask employees about it (Addition by M.H, "*this item was mentioned by some participants of the focus groups").*
- The experience of loneliness by means of scale items is difficult or impossible to get out of people with dementia

#### 2.1.4.2. Considerations regarding operationalization and definitions

- The JGS was developed at the time on the basis of open interviews to experiences (bottom to top). This presented scale development is based on "top to bottom", on predefined concepts, in which lived experiences are sought. The content of the concepts and dimensions must then be properly described.
- It is a choice what you consider loneliness. There is a stretching of the concept of loneliness. Related to this, it is questionable whether the items presented such as *powerlessness and meaninglessness* still fall under the definition of loneliness
- Loneliness is measured by the realization of individual and social desires/needs, "the desire for" is not loneliness.
- Causal factors such as "not being seen" are not the same as the person's experiences of loneliness. The items of the well-known loneliness scales are a mix of situations, experience and feelings, which articulate a person's reflection on a certain situation not the causes
- There are objections to measuring items separately such as "what are you going to measure?" People have so many different contexts and frames of reference. There are more steps to be taken when focusing on dementia.

#### 2.1.4.3. Considerations regarding the distinction in dimensions of loneliness

- The definition of emotional loneliness is less strongly defined than that of loneliness itself. Empirically, emotional and social loneliness correlate strongly, distinction can occur in situations.
- The dimensions are in connection with each other: It is not 1 or other, but a layering, so levels: As an example, the 4 layers of meaning (Carlo Legett); factual, emotional, biographical and existential.
- The individual scale items thus touch each other, e.g. the lack of meaningful relationships is also related to existential loneliness. Social loneliness thus touches on identity and thus on existence. Care workers should be able to recognize this layering (analogous to the training courses on meaning in <name nursing home>.

#### 2.1.4.4. Considerations for Scale Item and Content

- The current scale items are, in conjunction, the most representative of the experience of loneliness, demarcation or isolation of items separately to loneliness is difficult.
- It is necessary to maintain sufficient broadness within the concept of loneliness (by using all scale items), narrowing is particularly risky within the versatility of the nursing home population.
- Instead of reducing, better concretizing items**.**
- "Feeling empty" is a connecting item that reflects the experience of loneliness.

#### 2.1.4.5. After the development of a scale

- Try out the instrument carefully, don't use it immediately for comparisons.
- An instrument for rapid assessment (for research) is not compatible with the practical assessment scale for loneliness in care practice among individual residents. (Which can be used by care workers when loneliness is suspected).

#### 2.1.4.6. Additional Suggestions

- Selecting items by means of factor analysis of research (Schoenmakers & Tromp, 2022). The risk here is a distortion of the picture (more distinction than in reality, selecting on dependent variation is risky in scientific research).
- Or base it on the experiences of residents of people with dementia, (such as not feeling seen and heard") "Loading the understanding and from there doing the operationalization"
- Traditional line: Based on existing scales.
  a. Getting a grip on existing scales by means of "Thinking out loud protocol".
  b. Ask questions and let them think out loud. (Alternative 🡪 submitting existing items and doing psychometric research afterwards)
- More modestly, first the fullness of the concept of loneliness in the vph. Experiencing the setting/"loading"
- Look at the approach to the development of scale in people with intellectual disabilities

## 2.2. Institutional aspects a result of secondary analysis.

| **Lone-**  **liness** | **Base** | **Original wording** | **Quotes of selected studies** |
| --- | --- | --- | --- |
| Social | JGS 1 | *There is always someone I can talk to about my day-to-day problems* | *"aloneness"* [7:6 ¶ 3 in Jansson et al.  (2022)](https://go.atlasti.com/32acf5ab-c158-49e2-b0a0-b49ecbae0383/documents/022a607d-2c58-4d33-ab8d-e28c39a10020/quotations/3eff312a-c39f-402e-bfe3-cf19cd27bd84)  *"Being unable to reach out due to physical or mental impairment"* [6:5 ¶ 9 in Huang et al. (2022)](https://go.atlasti.com/32acf5ab-c158-49e2-b0a0-b49ecbae0383/documents/aac39f1d-a8f2-446d-a662-d3f54957a6af/quotations/971b9329-8bbd-4a40-ba83-09ea8a4eae2b)  *"Being alone"* [6:7 ¶ 11 in Huang et al. (2022)](https://go.atlasti.com/32acf5ab-c158-49e2-b0a0-b49ecbae0383/documents/aac39f1d-a8f2-446d-a662-d3f54957a6af/quotations/9b2c6c75-d6b0-4c81-8751-e7cd0b8cfc7c)  *"an absence of companions to talk to everyday"* [1:3 ¶ 6 in Amzat & Jayawardena (2016)](https://go.atlasti.com/32acf5ab-c158-49e2-b0a0-b49ecbae0383/documents/d5722ecd-6bfc-4ca0-9ba0-9e71857db695/quotations/27b4e9cb-b333-49e8-bd00-661152aa7d7d)  *"lack of social interaction"* [7:2 ¶ 3 in Jansson et al.  (2022)](https://go.atlasti.com/32acf5ab-c158-49e2-b0a0-b49ecbae0383/documents/022a607d-2c58-4d33-ab8d-e28c39a10020/quotations/09c3030a-3dd2-4448-93d9-f0b80286b263)  *"not have access to meaningful social interaction, whether with a partner, children, broader family, friends, fellow residents, or staff"* [2:2 ¶ 7 in Barbosa Neves et al. (2019)](https://go.atlasti.com/32acf5ab-c158-49e2-b0a0-b49ecbae0383/documents/260d6654-eae1-4c3c-ae24-eedeac472ff2/quotations/6387d1f0-5bf1-4cfc-822c-9518b11583f0)  *" few relationships"* [7:1 ¶ 3 in Jansson et al.  (2022)](https://go.atlasti.com/32acf5ab-c158-49e2-b0a0-b49ecbae0383/documents/022a607d-2c58-4d33-ab8d-e28c39a10020/quotations/6225310b-994d-4c95-a619-695c215c0d38)  *"individualization (in social activities),"* [2:16 ¶ 23 in Barbosa Neves et al. (2019)](https://go.atlasti.com/32acf5ab-c158-49e2-b0a0-b49ecbae0383/documents/260d6654-eae1-4c3c-ae24-eedeac472ff2/quotations/9c85f667-09c7-40c4-996d-29a0dee84f67)  *"loss of cloas (spousal and familiy)"* [2:1 ¶ 6 in Barbosa Neves et al. (2019)](https://go.atlasti.com/32acf5ab-c158-49e2-b0a0-b49ecbae0383/documents/260d6654-eae1-4c3c-ae24-eedeac472ff2/quotations/9316d8f2-83e7-4634-a306-7a483b77216f)  *"Being disconnected from past social interactions"* [6:2 ¶ 4 in Huang et al. (2022)](https://go.atlasti.com/32acf5ab-c158-49e2-b0a0-b49ecbae0383/documents/aac39f1d-a8f2-446d-a662-d3f54957a6af/quotations/ed73fd92-fd7a-4473-82cd-c446d0a207c2)  *"A lack of family and friends"* [9:2 ¶ 7 in Paque et al. (2018)](https://go.atlasti.com/32acf5ab-c158-49e2-b0a0-b49ecbae0383/documents/ef15ab07-a885-4e15-8edd-aea8c9d9503b/quotations/dcdc8218-abbb-4c94-b49d-e92b37b72f18)  *"loss of friends, and fear of making new friends and losing them"* [2:10 ¶ 16 in Barbosa Neves et al. (2019)](https://go.atlasti.com/32acf5ab-c158-49e2-b0a0-b49ecbae0383/documents/260d6654-eae1-4c3c-ae24-eedeac472ff2/quotations/3ba2920b-cefd-43e8-a4b0-45ac0ccbab91)  *"Being self-isolated due to feelings of inferiority"* [6:6 ¶ 10 in Huang et al. (2022)](https://go.atlasti.com/32acf5ab-c158-49e2-b0a0-b49ecbae0383/documents/aac39f1d-a8f2-446d-a662-d3f54957a6af/quotations/39484035-4cf0-4e5b-88d4-2a74b90dcb73)  *"Relationships with important others did not meet the participants’ expectations"* [9:3 ¶ 8 in Paque et al. (2018)](https://go.atlasti.com/32acf5ab-c158-49e2-b0a0-b49ecbae0383/documents/ef15ab07-a885-4e15-8edd-aea8c9d9503b/quotations/c2142230-25e6-4c92-806f-395e9d6b8bb3)  *"the quantity of the visits and the quality of the conversations is unsatisfactory"* [9:4 ¶ 9 in Paque et al. (2018)](https://go.atlasti.com/32acf5ab-c158-49e2-b0a0-b49ecbae0383/documents/ef15ab07-a885-4e15-8edd-aea8c9d9503b/quotations/df4b085d-e56b-4212-8ada-4181238c16d1)  *"missing company"* [7:5 ¶ 3 in Jansson et al.  (2022)](https://go.atlasti.com/32acf5ab-c158-49e2-b0a0-b49ecbae0383/documents/022a607d-2c58-4d33-ab8d-e28c39a10020/quotations/2c9f1714-27ee-4206-8ce2-98e22f782cc8)  *"not sharing and processing negative feelings"* [2:14 ¶ 21 in Barbosa Neves et al. (2019)](https://go.atlasti.com/32acf5ab-c158-49e2-b0a0-b49ecbae0383/documents/260d6654-eae1-4c3c-ae24-eedeac472ff2/quotations/69f48555-6974-4cee-b63d-723354712a4c).  *"not having made enough friends"* [2:12 ¶ 19 in Barbosa Neves et al. (2019)](https://go.atlasti.com/32acf5ab-c158-49e2-b0a0-b49ecbae0383/documents/260d6654-eae1-4c3c-ae24-eedeac472ff2/quotations/1b1ff5b8-cedf-4229-9f98-04d938c3280d) |
|  | JGS 4 /SJGS 2 | *There are plenty of people I can lean on when I have problems* |  |
|  | JGS 8  SJGS 4 | *There are enough people I feel close to* |  |
|  | JGS 11 | *I can call on my friends whenever I need them* |  |
|  | JGS 7  /SJGS 3 | *There are many people I can trust completely* |  |
| Emoti-  onal | JGS 2 | *I miss having a really close friend* | *"longing for a meaningful other"* [7:7 ¶ 4 in Jansson et al.  (2022)](https://go.atlasti.com/32acf5ab-c158-49e2-b0a0-b49ecbae0383/documents/022a607d-2c58-4d33-ab8d-e28c39a10020/quotations/fbdd7d13-02b7-4b69-80d9-07ec928d90de)  *"nothingness"* [7:11 ¶ 5 in Jansson et al.  (2022)](https://go.atlasti.com/32acf5ab-c158-49e2-b0a0-b49ecbae0383/documents/022a607d-2c58-4d33-ab8d-e28c39a10020/quotations/c25e5b23-a9bc-4afe-a7a7-cd08369ac79b)  *"mundane loneliness"* [7:9 ¶ 4 in Jansson et al.  (2022)](https://go.atlasti.com/32acf5ab-c158-49e2-b0a0-b49ecbae0383/documents/022a607d-2c58-4d33-ab8d-e28c39a10020/quotations/1c47fdd0-fde6-40a5-a04a-f1462626faf6)  *"Experience of isolation"* [3:3 ¶ 7 in Chung et al. (2020)](https://go.atlasti.com/32acf5ab-c158-49e2-b0a0-b49ecbae0383/documents/2f568102-37ea-46d9-9dff-103d1cd0701e/quotations/6ee23cd8-629c-484d-adcd-84a9ee99545c)  *"the painful loss of important people"* [10:1 ¶ 3, El: the painful loss of important people in Sundström et al. (2019)](https://go.atlasti.com/32acf5ab-c158-49e2-b0a0-b49ecbae0383/documents/8f3c56ea-40a3-4636-8f26-2407ff1da90f/quotations/31dd7ef0-edf2-4a86-bc36-cabf6bce8d7d)  *"no one to talk with"* [1:7 ¶ 11 in Amzat & Jayawardena (2016)](https://go.atlasti.com/32acf5ab-c158-49e2-b0a0-b49ecbae0383/documents/d5722ecd-6bfc-4ca0-9ba0-9e71857db695/quotations/9cba30a8-ecff-4fe1-8f18-ab3ad49a64fe)  *"Having no family or seldom visits of family"* [1:13 ¶ 17 in Amzat & Jayawardena (2016)](https://go.atlasti.com/32acf5ab-c158-49e2-b0a0-b49ecbae0383/documents/d5722ecd-6bfc-4ca0-9ba0-9e71857db695/quotations/bdfd3101-329f-4d4a-a011-c1f9275f9417)  *"the lack of social or emotional support of their family"* [1:12 ¶ 16 in Amzat & Jayawardena (2016)](https://go.atlasti.com/32acf5ab-c158-49e2-b0a0-b49ecbae0383/documents/d5722ecd-6bfc-4ca0-9ba0-9e71857db695/quotations/dea893ba-4e37-43d1-bf5b-22048ccb41bb)  *"poor relationships between older people and their relatives,"* [10:2 ¶ 4 in Sundström et al. (2019)](https://go.atlasti.com/32acf5ab-c158-49e2-b0a0-b49ecbae0383/documents/8f3c56ea-40a3-4636-8f26-2407ff1da90f/quotations/4317475d-9993-4e88-b20d-6a981ac38154)  *"the expectations of the residents towards their families and those close to them"* [1:10 ¶ 14 in Amzat & Jayawardena (2016)](https://go.atlasti.com/32acf5ab-c158-49e2-b0a0-b49ecbae0383/documents/d5722ecd-6bfc-4ca0-9ba0-9e71857db695/quotations/dcd606bf-0629-4612-b10a-d05a8b4bee31)  *"having no one there for them either physically or emotionally"* [1:2 ¶ 5 in Amzat & Jayawardena (2016)](https://go.atlasti.com/32acf5ab-c158-49e2-b0a0-b49ecbae0383/documents/d5722ecd-6bfc-4ca0-9ba0-9e71857db695/quotations/a880acf4-f6c1-4740-8b4a-a0480bd4bc1d)  *"Endlessly waiting for a family visit"* [6:1 ¶ 3 in Huang et al. (2022)](https://go.atlasti.com/32acf5ab-c158-49e2-b0a0-b49ecbae0383/documents/aac39f1d-a8f2-446d-a662-d3f54957a6af/quotations/4bf69a83-77d9-444d-9bca-95e57b350d03)  *"social death"* [7:14 ¶ 5 in Jansson et al.  (2022)](https://go.atlasti.com/32acf5ab-c158-49e2-b0a0-b49ecbae0383/documents/022a607d-2c58-4d33-ab8d-e28c39a10020/quotations/a7cdc80e-bba3-4379-9587-786383f77530)  *"Failure to be understood"* [3:2 ¶ 5 in Chung et al. (2020)](https://go.atlasti.com/32acf5ab-c158-49e2-b0a0-b49ecbae0383/documents/2f568102-37ea-46d9-9dff-103d1cd0701e/quotations/2330acd7-10c4-4a05-8cb2-6e11ddfb8915)  *"neglect and abandonment"* [1:4 ¶ 7 in Amzat & Jayawardena (2016)](https://go.atlasti.com/32acf5ab-c158-49e2-b0a0-b49ecbae0383/documents/d5722ecd-6bfc-4ca0-9ba0-9e71857db695/quotations/90126eb5-b613-4c77-be84-e34068531112)  *"feelings of broader social abandonment and rejection because of being old"* [2:8 ¶ 14 in Barbosa Neves et al. (2019)](https://go.atlasti.com/32acf5ab-c158-49e2-b0a0-b49ecbae0383/documents/260d6654-eae1-4c3c-ae24-eedeac472ff2/quotations/6e68ca33-c389-4946-a061-e0c41ed25955)  *"social exclusion"* [7:4 ¶ 3 in Jansson et al.  (2022)](https://go.atlasti.com/32acf5ab-c158-49e2-b0a0-b49ecbae0383/documents/022a607d-2c58-4d33-ab8d-e28c39a10020/quotations/02a742af-2270-41a8-906a-9ee000592c3e)  *"feeling of being left out and unappreciated"* [9:1 ¶ 6 in Paque et al. (2018)](https://go.atlasti.com/32acf5ab-c158-49e2-b0a0-b49ecbae0383/documents/ef15ab07-a885-4e15-8edd-aea8c9d9503b/quotations/c91943b6-c8f2-42a8-9d06-e82b372ef542)  *"worried, frustrated, and depressed by disruption of familiar roles and life goals"* [3:5 ¶ 10 in Chung et al. (2020)](https://go.atlasti.com/32acf5ab-c158-49e2-b0a0-b49ecbae0383/documents/2f568102-37ea-46d9-9dff-103d1cd0701e/quotations/c414b558-4e57-4a6b-8ab2-8923a82c4b06)  *"Feeling offended"* [8:2 ¶ 5, Ex: Feeling offended in Österlind, et al. (2017)](https://go.atlasti.com/32acf5ab-c158-49e2-b0a0-b49ecbae0383/documents/af79cb8c-0fdb-4696-a5a1-a0b98da91723/quotations/25460c28-c2a0-478b-b8e9-b0a5cbc476b3) |
|  | JGS 3  SJGS 1 | *I experience a general sense of emptiness* |  |
|  | JGS 5 | *I miss the pleasure of the company of other* |  |
|  | JGS-6 | *I find my circle of friends and acquaintances too limited* |  |
|  | JGS-9  /SJGS 5 | *I miss having people around me* |  |
|  | JGS-10  /SJGS 6 | *I often feel rejected* |  |
| Exis-  tential | ELQ 1 | *I am happy with the way I have lived my life* |  |
|  | ELQ 3 | *There is a purpose to my life* | *"useless because they themselves and others assumed they could no longer contribute to life"* [3:7 ¶ 12 in Chung et al. (2020)](https://go.atlasti.com/32acf5ab-c158-49e2-b0a0-b49ecbae0383/documents/2f568102-37ea-46d9-9dff-103d1cd0701e/quotations/48613178-199b-4c27-9b3e-21ddec17ec47)  *"loss of feeling functionally significant"* [5:3 ¶ 7 in Ho et al. (2022)](https://go.atlasti.com/32acf5ab-c158-49e2-b0a0-b49ecbae0383/documents/481db47c-65a0-4e05-aa16-a9ebf75dd41e/quotations/59938ae2-dd05-4466-b3b8-7e4945aad273)  *"Perceptions “thinking negatively on their situation"* [1:11 ¶ 15 in Amzat & Jayawardena (2016)](https://go.atlasti.com/32acf5ab-c158-49e2-b0a0-b49ecbae0383/documents/d5722ecd-6bfc-4ca0-9ba0-9e71857db695/quotations/49919652-d1b6-4c7c-b442-ded74ba2f624) |
|  | ELQ18 | *I feel helpless* | *"deserted and helpless because the experience of ExL led to emotional instability, insecurity, and negativity"* [3:8 ¶ 13 in Chung et al. (2020)](https://go.atlasti.com/32acf5ab-c158-49e2-b0a0-b49ecbae0383/documents/2f568102-37ea-46d9-9dff-103d1cd0701e/quotations/cce63169-9c9a-496b-a4fa-8174b1b12082)  *"Feeling upset, depressed, hopeless, and suffering"* [6:3 ¶ 6 in Huang et al. (2022)](https://go.atlasti.com/32acf5ab-c158-49e2-b0a0-b49ecbae0383/documents/aac39f1d-a8f2-446d-a662-d3f54957a6af/quotations/ba15d71f-66ff-4200-afb2-8b3e8d5446a9)  *"feelings of grief caused by losing autonomy and other loss"* [9:9 ¶ 15 in Paque et al. (2018)](https://go.atlasti.com/32acf5ab-c158-49e2-b0a0-b49ecbae0383/documents/ef15ab07-a885-4e15-8edd-aea8c9d9503b/quotations/7c9fc9b8-e963-42b4-9a25-466361c17ef0) |
|  | ELQ25 | *I feel at the mercy of the world* | *"a significant loss of autonomy due to living in an NH"* [9:7 ¶ 13 in Paque et al. (2018)](https://go.atlasti.com/32acf5ab-c158-49e2-b0a0-b49ecbae0383/documents/ef15ab07-a885-4e15-8edd-aea8c9d9503b/quotations/a52d3e92-0905-431a-9c3c-1303e838ec76)  *"Loss of control of life"* [3:1 ¶ 4 in Chung et al. (2020)](https://go.atlasti.com/32acf5ab-c158-49e2-b0a0-b49ecbae0383/documents/2f568102-37ea-46d9-9dff-103d1cd0701e/quotations/475a87f9-cf49-492e-9cbd-59d62173eec1)  *"the loss of autonomy due to physical deterioration"* [9:8 ¶ 14 in Paque et al. (2018)](https://go.atlasti.com/32acf5ab-c158-49e2-b0a0-b49ecbae0383/documents/ef15ab07-a885-4e15-8edd-aea8c9d9503b/quotations/d23f49fa-23e1-4b94-91d3-d9db7cf4ed7c) |
|  | ELQ26 | *I feel dead* | *"residents approaching death"* [10:4 ¶ 6 in Sundström et al. (2019)](https://go.atlasti.com/32acf5ab-c158-49e2-b0a0-b49ecbae0383/documents/8f3c56ea-40a3-4636-8f26-2407ff1da90f/quotations/038f0b88-13b1-47de-94d9-637a2961db8a)  *"thinking of oneself as dead"* [8:1 ¶ 5 in Österlind, et al. (2017)](https://go.atlasti.com/32acf5ab-c158-49e2-b0a0-b49ecbae0383/documents/af79cb8c-0fdb-4696-a5a1-a0b98da91723/quotations/e71dc220-f05c-42c4-8e29-305aaa4ce6fe) |
|  | ELQ27 | *The universe is full of meaning* | *"‘leading a meaningless life"*  [9:11 ¶ 17 in Paque et al. (2018)](https://go.atlasti.com/32acf5ab-c158-49e2-b0a0-b49ecbae0383/documents/ef15ab07-a885-4e15-8edd-aea8c9d9503b/quotations/e68458f3-ad91-4ac7-8c9c-e622fc05f4a7)  *"Inability to identify meaning of life"* [3:4 ¶ 8 in Chung et al. (2020)](https://go.atlasti.com/32acf5ab-c158-49e2-b0a0-b49ecbae0383/documents/2f568102-37ea-46d9-9dff-103d1cd0701e/quotations/8f715ccb-7bf6-435e-b794-f204b115b0f8)  *"meaningless life"* [7:8 ¶ 4 in Jansson et al.  (2022)](https://go.atlasti.com/32acf5ab-c158-49e2-b0a0-b49ecbae0383/documents/022a607d-2c58-4d33-ab8d-e28c39a10020/quotations/017f374d-ed31-4bd8-962a-7811d7e5c303) |
|  | ELQ29 | *I feel that there is little point to life* | *"Facing death and lonely humanity"* [7:13 ¶ 5 in Jansson et al.  (2022)](https://go.atlasti.com/32acf5ab-c158-49e2-b0a0-b49ecbae0383/documents/022a607d-2c58-4d33-ab8d-e28c39a10020/quotations/3a69fb67-93ee-410a-af72-7e6e0e866093)  *"Lonely waiting"* [7:12 ¶ 5 in Jansson et al.  (2022)](https://go.atlasti.com/32acf5ab-c158-49e2-b0a0-b49ecbae0383/documents/022a607d-2c58-4d33-ab8d-e28c39a10020/quotations/e34b5d05-955d-4759-83be-d4f7f563067d)  *"disconnection with prior commitments"* [5:1 ¶ 5 in Ho et al. (2022)](https://go.atlasti.com/32acf5ab-c158-49e2-b0a0-b49ecbae0383/documents/481db47c-65a0-4e05-aa16-a9ebf75dd41e/quotations/3d2ddf1d-faf4-4376-b5bc-db372df0b9db)  *"desperation due to recognition that their life is near its end"* [3:6 ¶ 11 in Chung et al. (2020)](https://go.atlasti.com/32acf5ab-c158-49e2-b0a0-b49ecbae0383/documents/2f568102-37ea-46d9-9dff-103d1cd0701e/quotations/32ca8d64-0fc6-4774-81d9-4e47e8566b7d) |
| Insti-  tional  Aspects  of  Lone-liness | RA1=  IAL 1 | *I lose my identity*  *(Lacking self identity)* | *"Routinization (related to living in care-homes)"* [2:18 ¶ 26 in Barbosa Neves et al. (2019)](https://go.atlasti.com/32acf5ab-c158-49e2-b0a0-b49ecbae0383/documents/260d6654-eae1-4c3c-ae24-eedeac472ff2/quotations/b1bc878e-04fd-4b37-b0f9-a2214888b9da)  *"a collapse in self-interpretation"* [5:2 ¶ 6 in Ho et al. (2022)](https://go.atlasti.com/32acf5ab-c158-49e2-b0a0-b49ecbae0383/documents/481db47c-65a0-4e05-aa16-a9ebf75dd41e/quotations/826be5a1-2f18-474b-a294-bd2f09c957e4)  *"prisoner of loneliness"* [7:15 ¶ 5 in Jansson et al.  (2022)](https://go.atlasti.com/32acf5ab-c158-49e2-b0a0-b49ecbae0383/documents/022a607d-2c58-4d33-ab8d-e28c39a10020/quotations/a55af755-2f45-463f-8bb8-33dfa5ef059d)  *"Lacking self-identity: (they could only play the role of individuals who need to be taken care of by others)"* [6:9 ¶ 14 in Huang et al. (2022)](https://go.atlasti.com/32acf5ab-c158-49e2-b0a0-b49ecbae0383/documents/aac39f1d-a8f2-446d-a662-d3f54957a6af/quotations/868afd34-bf99-49eb-8754-d55d28101c68) |
|  | RA 3 =  IAL 2 | *I feel like I belong  (lacking a sense of place)* | *"feeling like a stranger in the care unit"* [10:3 ¶ 5 in Sundström et al. (2019)](https://go.atlasti.com/32acf5ab-c158-49e2-b0a0-b49ecbae0383/documents/8f3c56ea-40a3-4636-8f26-2407ff1da90f/quotations/3068831e-a4c4-48c0-843c-736999ae5959)  *"Feeling trapped"* [8:3 ¶ 5 in Österlind, et al. (2017)](https://go.atlasti.com/32acf5ab-c158-49e2-b0a0-b49ecbae0383/documents/af79cb8c-0fdb-4696-a5a1-a0b98da91723/quotations/d46855ce-de09-4b5d-82f4-f9f616f6ff30)  *"Lacking a sense of place"* [6:8 ¶ 13 in Huang et al. (2022)](https://go.atlasti.com/32acf5ab-c158-49e2-b0a0-b49ecbae0383/documents/aac39f1d-a8f2-446d-a662-d3f54957a6af/quotations/43757446-3e3e-4da8-9e44-208d85e9c9bd) |
|  | RA 4 IAL 3 | *I often have a strong desire for the past (reminiscence)* | *"feeling sad and lonely when thinking about their past"* [1:9 ¶ 13 in Amzat & Jayawardena (2016)](https://go.atlasti.com/32acf5ab-c158-49e2-b0a0-b49ecbae0383/documents/d5722ecd-6bfc-4ca0-9ba0-9e71857db695/quotations/240e64af-40d4-4af6-995e-63684c645a3f)  *"reminiscencing of a younger past"* [2:4 ¶ 10 in Barbosa Neves et al. (2019)](https://go.atlasti.com/32acf5ab-c158-49e2-b0a0-b49ecbae0383/documents/260d6654-eae1-4c3c-ae24-eedeac472ff2/quotations/b8685b7c-061e-4897-b54d-464389f92066)  *"expression of a strong desire to go back home or restore their old lives"* [9:10 ¶ 16 in Paque et al. (2018)](https://go.atlasti.com/32acf5ab-c158-49e2-b0a0-b49ecbae0383/documents/ef15ab07-a885-4e15-8edd-aea8c9d9503b/quotations/98b925d1-5cc1-41bf-b9c4-d923d82cc418) |
|  | RA 2 | *feeling lonely in a group*  *(othering, being alone together)* | *"all alone in loneliness"* [7:10 ¶ 4 in Jansson et al.  (2022)](https://go.atlasti.com/32acf5ab-c158-49e2-b0a0-b49ecbae0383/documents/022a607d-2c58-4d33-ab8d-e28c39a10020/quotations/f6ab754b-8b2d-4f63-b909-17251457dc1f)  *lonely in a crowd"* [7:3 ¶ 3 in Jansson et al.  (2022)](https://go.atlasti.com/32acf5ab-c158-49e2-b0a0-b49ecbae0383/documents/022a607d-2c58-4d33-ab8d-e28c39a10020/quotations/c712e9cc-d506-4d08-a902-42467db61c07)  *"alienation from other residents (othering, being ‘alone together’)"* [2:15 ¶ 22 in Barbosa Neves et al. (2019)](https://go.atlasti.com/32acf5ab-c158-49e2-b0a0-b49ecbae0383/documents/260d6654-eae1-4c3c-ae24-eedeac472ff2/quotations/6f476e83-a02e-4537-8e03-720dc1093e39) |

## 2.3 Evolution wording in expertgroups

**Date: 230814**

**Status: Conceptual**

| **Nr** | **Base** | **Englisch translation** | **Original Dutch wording** | **Simplified Statement ^[2]^** | **Approved question testscale ^[3]^** |
| --- | --- | --- | --- | --- | --- |
| **1** | **JGS 1** | There is always someone I can talk to about my day-to-day problems. | Er is altijd wel iemand in mijn omgeving bij wie ik met mijn dagelijkse probleempjes terecht kan. | *Als ik ergens mee zit en er over wilt praten, is er altijd een luisterend oor.* | Als u ***dagelijkse probleempjes*** *heeft, zijn er dan altijd mensen*  *met wie u kunt praten?* |
| **2** | **JGS 4*** | There are plenty of people I can lean on when I have problems. | Er zijn genoeg mensen op wie ik in geval van narigheid kan terugvallen. | *Als er echt nare dingen zijn, dan is er iemand die er voor mij is.* | Als u ***narigheid overkomt,*** zijn er dan genoeg mensen die u steunen? |
| **3** | **JGS 7*** | There are many people I can trust completely. | Ik heb veel mensen op wie ik volledig kan vertrouwen. | *Ik vertrouw volledig op de mensen om me heen.* | Zijn er *veel mensen* die u ***volledig*** *kunt* ***vertrouwen****?* |
| **4** | **JGS 8*** | There are enough people I feel close to | Er zijn voldoende mensen met wie ik me nauw verbonden voel. | *Er zijn genoeg mensen met wie ik een goede band heb.* | *Zijn er genoeg mensen met wie u* ***een goede band heeft?*** |
| **5** | **JGS 11** | I can call on my friends whenever I need them. | Wanneer ik daar behoefte aan heb kan ik altijd bij mijn vrienden terecht. | *M’n vrienden zijn er voor me al ik ze nodig heb.* | ***Kunt*** *u* ***bij uw vrienden terecht*** *als u dat nodig vindt?* |
| **6** | **JGS 2** | I miss having a really close friend. | Ik mis een echt goede vriend of vriendin. | *Wat ik hier mis is een maatje.* | *Mist u een* ***goede vriend of vriendin?*** |
| **7** | **JGS 3*** | I experience a general sense of emptiness. | Ik ervaar een leegte om me heen. | *Er is helemaal niets of niemand hier voor mij.* | ***Ervaart*** u een ***leegte*** om u heen*?* |
| **8** | **JGS 5** | I miss the pleasure of the company of other. | Ik mis gezelligheid om me heen. | *Het is hier een ongezellige boel.* | Mist u ***gezelligheid*** van anderen? |
| **9** | **JGS 6** | I find my circle of friends and acquaintances too limited. | Ik vind mijn kring van kennissen te beperkt. | *Ik ontmoet bijna nooit meer bekenden.* | Vindt u uw ***kring* van *kennissen*** te klein? |
| **10** | **JGS 9*** | I miss having people around me. | Ik mis mensen om me heen. | *Ik heb er last van dat er zo weinig mensen zijn die ik zie of spreek.* | *Mist u* ***mensen om u heen****?* |
| **11** | **JGS 10*** | I often feel rejected. | Vaak voel ik me in de steek gelaten. | *Ze laten me in de steek.* | ***Voelt*** *u zich vaak* ***in de steek gelaten****?* |
| **12** | **ELQ 1** | I am happy with the way I have lived my life. | Ik ben gelukkig over hoe ik mijn leven heb geleid. | *Het leven vroeger was goed.* | *Bent u tevreden over* ***hoe u*** ***heeft geleefd?*** |
| **13** | **ELQ 3** | There is a purpose to my life. | Mijn leven heeft een bepaalde bedoeling. | *Ik heb hier nog een doel.* | Heeft *uw* ***leven een doel?*** |
| **14** | **ELQ 18** | I feel helpless. | Ik voel me hulpeloos. | *Ik voel me zo hulpeloos.* | Voelt u zich ***hulpeloos?*** |
| **15** | **ELQ 25** | I feel at the mercy of the world. | Ik voel mij machteloos tegenover de wereld. | *Ik heb hier niets te vertellen.* | Voelt u zich ***machteloos tegenover de wereld****?* |
| **16** | **ELQ 26** | I feel dead. | Het voelt alsof ik dood ben. | *Ik doe er helemaal niet meer toe.* | Voelt u zich ***“Dood****” van binnen?* |
| **17** | **ELQ 27** | The universe is full of meaning. | Deze wereld biedt vele mogelijkheden. | *Er zijn hier veel mogelijkheden om zinvol bezig te zijn.* | Biedt deze wereld ***vele mogelijkheden?*** |
| **18** | **ELQ 29** | I feel that there is little point to life. | Het leven heeft weinig zin. | *Als ik hier moet leven dan heeft het geen zin.* | Heeft het *leven* ***weinig zin*** voor  u *?* |
| **19** | **IAL 1** | *I lose my identity.* | *Ik raak mijn eigenheid kwijt.* | *Ik kan mezelf (niet) zijn.* | *Bent u* ***uw eigenheid*** *kwijt?* |
| **20** | **IAL 2** | *I feel like I belong* | *Ik voel me thuis op deze plek* | *Ik voel me hier (niet) thuis* | *Voelt u zich hier op* ***uw plek?*** |
| **21** | **IAL 3** | *I often have a strong desire for the past* | *Ik heb vaak heimwee naar vroeger.* | *Ik wou dat ik weer terug kon naar vroeger* | *Heeft u vaak* ***heimwee naar vroeger****?* |
| **22** | **IAL 4** | *I feel alone, despite being together* | *Ik voel me buitengesloten van de andere bewoners.* | *Ik voel me buitengesloten* | *Voelt u zich* ***buiten-gesloten?*** |

Step 3 Pre-testing

## 3.1. Protocol Cognitive interviewing

|  | **Step** | | **Topic** | | **Description** | | **Time** | |
| --- | --- | --- | --- | --- | --- | --- | --- | --- |
| 1 | | Preparation | | - Prepare items, have pre-arranged set of questions ready for each respondent. | |  | |  |
| 2 | | Meeting the participant | | - Care staff introduces you to participant. - Start a conversation with them:   ask about how their day is going, something noticeable in the environment (weather, belongings, etc.) or whether the participant has any questions. | | 5-10 mins | |  |
| 3 | | Introducing the interview | | - Remind participant of the purpose of the visit and what is going to happen.   *“U helpt ons een goede vragenlijst op te stellen waarmee we eenzaamheid kunnen meten.”* | | 3-5 mins | |  |
| 4 | | Informed consent | | - Explain that the questions may be sensitive, participants can always decide to not answer a question. - Remind participant of audio recording, explain that the privacy of the participant will be respected. - Ask participant if they still agree to participate. | | 3-5 mins | |  |
| 5 | | Check before starting | | - Ask the participant if they feel ready to start. - If they still seem hesitant, ask them if there is anything that would make them feel more comfortable. | | 1-2 mins | |  |
| 6 | | Scale questions | | - Interviewer reads out the question, presents the 3 answer options and asks:   *“Welk antwoord zou u geven?”* | |  | |  |
| 7 | | General probing | | - Ask the participant some general probing questions:   *“Vond u het moeilijk of makkelijk deze vraag te beantwoorden?”* | |  | |  |
| 8 | | Example | | - If participant answers “*moeilijk*”, interviewer reads out example from item, asks if the participant wants to change their answer. | |  | |  |
| 9 | | Comprehension probing | | - Ask some comprehension probing questions:   *“Wat betekent het woord* ***(bolded in question)*** *in deze vraag voor u?”* | |  | |  |
| 10 | | Recall probing | | - Ask some recall probing questions:   *“Wanneer voelt u zich* ***(bolded in question)****?”* | |  | |  |
| 11 | | Check moving on | | - Once the participant is done answering, ask them if they are ready to move on to the next one. | |  | |  |
| 12 | | Repeat | | - Repeat steps 6-10, until about 30 mins have passed. | | 30 mins | |  |
| 13 | | Post interview check up | | - Ask the participant how they feel after the interview, and if there is anything else the participant would like to share.   *“Hoe is het met u na dit gesprek?”*  *“Wilt u nog wat delen met ons?”* | | 3-5 mins | |  |
| 14 | | Care staff | | - Ask care staff to come into the room and explain that the interview is finished. | | 1-2 mins | |  |
| 15 | | Further contact | | - Remind the participant and the care staff that they can contact the interviewer (via email) if they have any further questions or comments at any point. - Offer to keep them informed regarding the progress of the research, in order to keep them involved in the process. | | 1-2 mins | |  |
| 16 | | Give thanks | | - Remind the participant that their participation is greatly appreciated, and thank them for their time. | | 1-2 mins | |  |
| 17 | | Finish | | - Take some time to finish off the conversation in a comfortable way. | | 5-10 mins | |  |
| 18 | | Care staff | | - Once you or the participant has left, talk to care staff in charge and ask them to check in with the participant after a few days. | | 3-5 mins | |  |

1. The items as applied by Schoenmakers & Tromp , (11 item DJG, and 8 items from the ELQ + direct question)	(2022)
2. Result of multidisciplinary expert group consultation (language and dementia specialist)
3. Result of review of panel of older adults and final review in research team
